# Comparative Genomics Provide Insights Into Karyotype Evolution in Vespertilionid Bats (Vespertilionidae, Chiroptera)

**DOI:** 10.1111/1755-0998.70129

**Published:** 2026-03-20

**Authors:** Linjing Lan, Xin Zhang, Jinjin Xie, Xiaohui Lin, Xihong Hong, Wenhui Nie, Jinhuan Wang, Weiting Su, Fengtang Yang, Guimei He, Xiuguang Mao

**Affiliations:** ^1^ School of Ecological and Environmental Sciences East China Normal University Shanghai China; ^2^ State Key Laboratory of Genetic Resources and Evolution Kunming Institute of Zoology, the Chinese Academy of Sciences Kunming Yunnan China; ^3^ School of Life Sciences and Medicine Shandong University of Technology Zibo China; ^4^ School of Life Science East China Normal University Shanghai China; ^5^ Institute of Eco‐Chongming (IEC) East China Normal University Shanghai China; ^6^ Shanghai Institute of Wildlife Epidemics East China Normal University Shanghai China

**Keywords:** bats, chromosomal evolution, comparative genomics, genome evolution, phylogeny

## Abstract

Studies elucidating the molecular basis and evolutionary consequences of karyotypic changes in mammals remain scarce. Here, we investigate chromosomal evolution by focusing on two contrasting lineages within the family Vespertilionidae (Chiroptera): the karyotypically variable tribe Pipistrellini and the highly conserved genus *Myotis*. Pipistrellini exhibits extensive karyotype diversity, with diploid numbers (2n) ranging from 26 to 44, whereas *Myotis* demonstrates remarkable stability, maintaining 2n = 44 across nearly all studied species. To uncover the mechanisms driving these divergent evolutionary trajectories, we generated a high‐quality chromosome‐level genome assembly for *Pipistrellus abramus* (2n = 26). By integrating multiple high‐quality vespertilionid genomes, we reconstructed the family phylogeny and inferred an ancestral karyotype of 2n = 44, revealing fusions and fissions as the primary drivers of karyotypic diversification. We further identified an enrichment of rolling‐circle (RC) and recent DNA transposons in genes involved in DNA metabolism, suggesting a mechanistic basis for transposable element (TE) tolerance in Vespertilionidae. In *P. abramus* , a derived chromosome originated from three ancestral chromosomes via Robertsonian and end‐to‐end fusions, with TEs significantly enriched at fusion sites. Genome stability‐related genes and contracted gene families also appear to facilitate adaptive responses to structural changes. These findings provide novel insights into the molecular mechanisms underlying chromosome evolution and speciation in mammals.

## 1 Introduction

Mammalian genome size exhibits remarkable diversity, spanning over five‐fold differences across species—from the compact 1.6 Gb genome of Carriker's round‐eared bat to the expansive 8.2 Gb genome of the red viscacha rat (Gregory 2025). The primary driver of this variation is differential accumulation of transposable elements (TEs), although other factors such as polyploidization and segmental duplication also contribute to changes in genome size (Chalopin et al. 2015; Canapa et al. 2016). Recent comparative analyses of > 200 eutherian genomes have quantitatively demonstrated a strong positive correlation between genome size expansion and TE accumulation (Osmanski et al. 2023). TEs proliferate within the genome through rapid self‐replication, a process that includes both “copy‐and paste” mechanisms for retrotransposons and “cut‐and‐paste” mechanisms for DNA transposons (Doolittle and Sapienza 1980). In addition, horizontal transfer (HT) of TEs across species represents another contributor to TE accumulation and, consequently, to the increase in genome size (Schaack et al. 2010; Wallau et al. 2018). For example, HT‐derived TEs contribute approximately 6% of the *Aedes* genome (Melo and Wallau 2020) and 2%–24% of the genome in various insect species (Peccoud et al. 2017).

Beyond expanding genome size, TEs profoundly influence genome architecture by facilitating chromosomal rearrangements, such as chromosome fusions, fissions, deletions, inversions, and translocations (Lönnig and Saedler 2002; Kidwell 2002; Eichler and Sankoff 2003; Brannan et al. 2023). Among these, chromosome fusions and fissions are the primary mechanisms underlying variations in the diploid chromosome number (2n) (Brannan et al. 2023). Such karyotypic shifts have been correlated with elevated diversification rates in several animal groups. For example, in Lepidoptera, chromosome fusions and fissions have been linked to rapid speciation (De Vos et al. 2020), and similar patterns are observed in the genus *Sceloporus* of lizards (Leaché et al. 2016). Mammals display particularly striking chromosome number variation, ranging from 2n = 6 in the female Indian muntjac to 2n = 102 in the red viscacha rat (Ferguson‐Smith and Trifonov 2007). Karyotypic restructuring is considered a major driver of speciation in various mammalian groups, such as rodents (e.g., Robertsonian fusions and fissions in the genus *Mus*; Romanenko et al. 2012), gibbons (Carbone et al. 2014), muntjac deer (Yin et al. 2021), and rock‐wallabies (*Petrogale*), where lineage‐specific fusions have facilitated ecological radiation (Potter et al. 2017). Inversions also play a crucial role in local adaptation and speciation by suppressing recombination within inverted regions, thereby resisting gene flow between lineages and maintaining genetic differences (Hoffmann and Rieseberg 2008; Faria et al. 2019; Wellenreuther and Bernatchez 2018).

Recent advances in third‐generation sequencing technologies, such as those offered by Oxford Nanopore and Pacific Biosciences, and high‐throughput chromosome conformation capture (Hi‐C) have enabled the construction of chromosome‐level genome assemblies, revolutionizing comparative genomics. These resources are increasing used to investigate genome size evolution, TEs dynamics, and the molecular underpinnings of diverse adaptive traits across mammals (Jebb et al. 2020; Blumer et al. 2022; Tian et al. 2023; Osmanski et al. 2023; Shao et al. 2023). Nevertheless, studies elucidating the molecular basis and evolutionary consequences of karyotypic changes in the mammalian lineage remain scarce, with few exceptions such as the work by Yin et al. (2021).

Bats (Chiroptera), constituting the second largest mammalian order with 1474 recognized species (Simmons and Cirranello 2025), are notable for possessing the smallest genome size among mammals. The average bat genome size is approximately 2.3 Gb, compared to ~3.4 Gb in other mammalian lineages (Smith et al. 2013; Gregory 2025). This notably compact genome size in bats is postulated to be a consequence of selective pressures imposed by the evolutionary adaptation to flight capabilities (Kapusta et al. 2017). Within bats, genome size exhibits approximately two‐fold variation, ranging from a low of ~1.6 Gb in Carriker's round‐eared bat (family Phyllostomidae) to a high of ~3.4 Gb in Hairy‐tailed bat (family Vespertilionidae) (Gregory 2025). A recent analysis of 37 bat genomes supported a linear correlation between genome size and TE content (Osmanski et al. 2023). Bats also represent a hotspot for HT of DNA transposons among mammals (Paulat et al. 2023). Notably, these HT events are predominantly observed in species belonging to the family Vespertilionidae, particularly those within the genus *Myotis*. To date, vespertilionid bats stand as the sole mammalian lineage that has been subjected to invasion by rolling‐circle (RC) transposons (Pritham and Feschotte 2007), a phenomenon that may have contributed to the family's diversification by promoting reproductive isolation through TE‐mediated genomic incompatibilities (Thomas et al. 2014). However, the evolutionary trajectory of TE accumulation and the impacts of specific TEs on genomic architecture in vespertilionid bats remain poorly understood.

The family Vespertilionidae exhibits exceptional karyotypic diversity, with 2n ranging from 18 to 52 across species (Sotero‐Caio et al. 2017). This wide variation provides unique opportunities to study contrasting evolutionary trajectories of chromosome evolution. For example, the genus *Myotis*, the most speciose chiropteran genus with 139 recognized species (Simmons and Cirranello 2025), demonstrates remarkable chromosomal conservation. Most *Myotis* species maintain a 2n = 44 karyotype through 21 million years of diversification, with only six exceptions (Bickham et al. 2004; Ruedi et al. 2013; reviewed in Vazquez et al. 2024). In striking contrast, the smaller genus *Pipistrellus* (33 species, Simmons and Cirranello 2025) displays a high degree of karyotypic variability, with nine different diploid numbers (2n = 26, 28, 30, 32, 34, 36, 38, 42, 44) (Pathak and Sharma 1969; Volleth et al. 2001). Recent phylogenetic studies supported the paraphyly of *Pipistrellus*, with a closer relationship between the “Eastern” *Pipistrellus* clade and *Glischropus*, and between the “Western” *Pipistrellus* clade and *Nyctalus* (Dool and Puechmaille 2025; Zhukova et al. 2025). Reflecting these updates, we adopt the tribe Pipistrellini, which includes 54 species, comprising *Pipistrellus*, *Glischropus*, *Nyctalus*, and three other genera (Simmons and Cirranello 2025). The contrasting karyotypic dynamics observed between Pipistrellini and *Myotis* lineages provide an ideal comparative framework for investigating molecular mechanisms underlying both extensive and conservative chromosomal evolution within a single family. A similar pattern has been reported in Lepidoptera, where most species exhibit stable karyotypes while certain lineages undergo frequent chromosomal changes (Wright et al. 2024). Unravelling the mechanisms behind such divergent evolutionary trajectories will enhance our understanding of chromosomal evolution and its contribution to speciation and adaptation across animals.

In this study, we generated a chromosome‐level genome assembly for *Pipistrellus abramus* , which possesses the lowest 2n in the genus *Pipistrellus* (2n = 26). This species commonly roosts in human‐made structures, particularly in cultivated lands such as paddy fields (Funakoshi and Uchida 1978). Additionally, *P. abramus* exhibits one of the smallest genome sizes in Chiroptera with an estimated size of 1.7 Gb (see Results). During the revision of this manuscript, 20 chromosome‐level vespertilionid genome assemblies have become available in the National Center for Biotechnology Information (NCBI) database. These genomes represent species from nine genera within Vespertilionidae (9/54 of the family), encompassing a wide range of diploid numbers (2n = 26, 28, 32, 38, 42, 44, 50). Leveraging these high‐quality genomic resources, we analysed genome size evolution across vespertilionid lineages and evaluate the contribution of TEs to genomic expansion. Using comparative genomics approaches, we reconstruct the ancestral karyotype of Vespertilionidae and identify key chromosomal rearrangements by comparing ancestral and extant karyotypes. We further explore the genetic mechanisms driving these rearrangements, with particular emphasis on the divergent evolutionary paths of Pipistrellini and *Myotis*.

## 2 Materials and Methods

### 2.1 Sampling and Sequencing

An adult male *Pipistrellus abramus* was collected in Chongming Island, Shanghai, China. After the bat was euthanized by cervical dislocation, eight fresh tissues (muscle, brain, cochlea, heart, liver, kidney, lung, and spleen) were carefully dissected and placed into RNase‐free tubes. All samples were immediately frozen in liquid nitrogen to prevent RNA degradation and stored at −80°C until further processing. The sampling and tissue collection procedures were approved by the National Animal Research Authority, East China Normal University (approval ID bf20190301), ensuring compliance with ethical guidelines for animal research.

Genomic DNA was extracted from muscle tissue using DNeasy kits (Qiagen). To achieve a high‐quality genome assembly, three complementary sequencing strategies were employed. First, a Nanopore long‐read library was prepared with DNA fragments longer than > 20 kb and sequenced on a PromethION sequencer (Oxford Nanopore). Raw Nanopore reads were quality‐assessed using Nanoplot v 1.40.2 (De Coster et al. 2018) and trimmed with Nanofilt v 2.8.0 (De Coster et al. 2018) using the parameters: ‐q 7 ‐l 1000 ‐‐headcrop 50 ‐‐tailcrop 50. Second, an Illumina short‐read library with an insert size of 350 bp was constructed and sequenced on the Illumina Novaseq 6000 platform (pair‐end 150 bp). These short reads were used for genome survey analysis and error correction of the Nanopore long reads. Third, a high‐throughput chromatin conformation capture (Hi‐C) library was constructed following the procedures described in previous study (Belton et al. 2012) to facilitate chromosome‐level scaffolding, and sequenced on the Novaseq platform (pair‐end 150 bp). Both Illumina short reads and Hi‐C reads were processed using fastp v 0.23.2 with default parameters (Chen et al. 2018).

For transcriptome sequencing, total RNA was extracted from each of the eight tissues using TRIzol (Life Technologies Corp. Carlsbad, CA, USA). Short‐read RNA sequencing libraries were constructed using NEBNext UltraTM RNA Library Prep Kit for Illumina (NEB, USA) and sequenced on the Novaseq platform (paired‐end, 150 bp). Raw RNA‐seq reads were trimmed using TRIMMOMATIC v 0.38 (Bolger et al. 2014) with the following parameters: SLIDINGWINDOW:4:20.

Detailed information about the genome and transcriptome sequencing data has been provided in Table S1.

### 2.2 Karyotype Analysis

To independently validate the chromosomal‐scale accuracy of our genome assembly, we performed karyotypic analysis on *P. abramus* specimens. This cytogenetic approach served as a critical benchmark for confirming chromosome number and morphology, thereby reinforcing the reliability of the reconstructed pseudochromosomes. Fibroblast cultures were established from bat ear biopsies. Metaphase chromosomes were prepared according to conventional procedures outlined by Nie et al. (2002). Karyotype analysis and the GTG‐banding were conducted following the standard protocol described by Seabright (1971).

### 2.3 Genome Assembly

Prior to de novo genome assembly, we performed a k‐mer‐based survey using 79.2 Gb of high‐quality Illumina short‐read data. The k‐mer spectrum was generated with Jellyfish v 2.2.10 (Marçais and Kingsford 2011) using a k‐mer size of 21, followed by genome characterization with GenomeScope v 2.0 (Vurture et al. 2017) to estimate genome size, heterozygosity and repeat sequence content. This initial survey provided essential parameters to guide the subsequent assembly process.

For contig‐level assembly, 165.55 Gb of trimmed Nanopore long reads were assembled using NextDenovo v 2.5.0 (accessible at https://github.com/Nextomics/NextDenovo) under the following parameters: read_cutoff = 10 k, pa_correction = 2, sort_options = −m 20 g ‐t 12 ‐k 50, correction_options = −p 10. The initial assembly underwent iterative polishing with Nextpolish v 1.4.1 (Hu et al. 2020) using both Nanopore and Illumina reads to enhance base‐level accuracy, followed by removal of redundant heterozygous contigs via Purge_Dups v 1.2.5 (Guan et al. 2020) to minimize assembly duplicates. Chromosomal‐scale scaffolding was achieved by integrating Hi‐C data. A total of 206.44 Gb of clean Hi‐C reads were aligned to the polished contigs using Juicer v 1.6 (Durand, Robinson, et al. 2016). The chromatin contact matrix was generated using the 3D‐DNA pipeline v 180,419 (Dudchenko et al. 2017) and subsequently manually curated in Juicebox Assembly Tools (Durand, Shamim, et al. 2016) with reference to the cytogenetically determined diploid chromosome number. This manual refinement involved adjusting chromosomal boundaries and orientations to ensure biological accuracy, significantly improving the assembly's reliability. The final chromosome‐level genome assembly was obtained through execution of the run‐ASM‐pipeline‐post‐review.sh script. Potential contaminant sequences were identified and removed using FCS‐GX (Astashyn et al. 2024) to ensure a clean reference genome.

The quality of the final genome assembly was evaluated through multiple approaches. First, completeness was assessed with Benchmarking Universal Single‐Copy Orthologs (BUSCO) v 5.2.2 (Simão et al. 2015) in genome mode against the mammalian database (mammalia_odb10). Second, we estimated the mapping rate through minimap2 v 2.24‐r1122 (Li 2018) for Nanopore long reads and bwa v 0.7.17‐r1188 (Li and Durbin 2010) for Illumina short reads and RNA‐seq reads. SAMtools v 1.16.1 (Danecek et al. 2021) was used to process the alignments. Third, base‐level accuracy was quantified using the Consensus Quality Value (QV) derived from Merqury v 1.3 (Rhie et al. 2020).

### 2.4 Genome Annotation

Our genome annotation pipeline integrated a combination of de novo and homology‐based strategies to ensure comprehensive and accurate identification of repetitive elements and protein‐coding genes. This multi‐pronged approach enhanced the reliability of annotations by leveraging both sequence similarity and structural features.

For repeat annotation, we employed a hybrid method that combined de novo prediction with database homology searches. A de novo repeat library was first reconstructed using RepeatModeler (accessible at https://github.com/Dfam‐consortium/RepeatModeler) with the ‘‐LTRStruct’ option. This library was then merged with existing resources, including a bat‐specific repeat library (Scheben et al. 2023, accessible at http://compgen.cshl.edu/bat/, comprising repeats from eight bat species), Repbase (accessible at http://www.girinst.org/repbase) and Dfam (accessible at http://dfam.org), to generate a comprehensive custom repeat library. Repeat sequences were annotated across the genome using RepeatMasker v 4.1.2 (Chen 2004) with the ‘‐xsmall ‐norna ‐poly’ option. To assess evolutionary dynamics, we calculated Kimura 2‐parameter distances between individual transposable element (TE) copies and their consensus sequences using the calcDivergenceFromAlign.pl. script within RepeatMasker, providing insights into TE activity and divergence.

Protein‐coding genes were predicted through three complementary approaches: ab initio, transcriptome‐based and homology‐based methods. First, the BRAKER2 v 2.5.2 pipeline (Brůna et al. 2021) was used to perform ab initio prediction. Second, transcriptome‐based prediction was conducted by mapping RNA‐seq data from eight tissues to the genome assembly using HISAT2 v 2.2.1 (Kim et al. 2015) with default parameters. Transcripts were assembled using StringTie v 2.2.1 (Pertea et al. 2015) in merge mode, and coding sequences were identified with TransDecoder v 5.5.0 (accessible at https://github.com/TransDecoder/TransDecoder) with default settings. Additionally, the PASA (Program to Assemble Spliced Alignments) pipeline v 2.5.2 (Haas et al. 2003) was used to align RNA‐seq reads and assemble transcript‐based gene models. Third, homology‐based prediction was carried out with GEMOMA v 1.9.0 (Keilwagen et al. 2016, 2018) using protein sequences from 12 evolutionarily diverse species (Table S2). All gene models were integrated and refined using EVidenceModeler v 2.0.0 (Haas et al. 2008) with a weighted consensus strategy (ABINITIO_PREDICTION AUGUSTUS 1, ABINITIO_PREDICTION GeneMark.hmm3 1, TRANSCRIPT Cufflinks 12, OTHER_PREDICTION GeMoMa 10, OTHER_PREDICTION transdecoder 12). This consolidation ensured high‐confidence gene structures by reconciling predictions from multiple sources. Functional annotation of protein‐coding genes was performed through BLASTP searches against the UniProt and non‐redundant (NR) databases using DIAMOND (e‐value = 1e‐5) supplemented by functional categorization and orthology assignment with eggNOG‐Mapper.

Sex chromosome identification relied on synteny and conserved gene content. The X chromosome was delineated through syntenic alignment with the *Myotis myotis* genome (Jebb et al. 2020), while the Y chromosome was detected via BLASTn searches for conserved Y‐linked genes, such as EIF1AY (Godfrey et al. 2020).

### 2.5 Mitochondrial Genome Assembly

In addition to nuclear genome assembly, we assembled a complete mitochondrial genome from Illumina short reads using MitoZ v.3.3 (Meng et al. 2019). The resulting assembly exhibited 99.41% sequence identity with the previously published mitochondrial genome of this species (GenBank accession: KX355640; Kim, Farré, et al. 2017) (Table S3 and Figure S1).

### 2.6 Phylogenetic Analysis and Divergence Time Estimation

To reconstruct the evolutionary relationships and divergence times within Vespertilionidae, we integrated genomic data from multiple sources. In addition to the *P. abramus* genome generated in this study, we included 20 chromosome‐level vespertilionid genome assemblies and five outgroups ( *Rousettus aegyptiacus* , *Rhinolophus ferrumequinum* , *Phyllostomus discolor* , *Desmodus rotundus* , *Molossus molossus* ) derived from the NCBI database (Table S4). To improve taxonomic representation, we further incorporated eight high‐quality contig‐level genome assemblies from Liu et al. (2024), ensuring coverage of all four subfamilies of Vespertilionidae. In total, our dataset included species from 16 out of the 54 genera within the family (Table S4), providing a comprehensive phylogenetic framework.

Protein sequences for eight species were directly downloaded from NCBI (Table S4). For the remaining species, we used a homology‐based method for gene annotation, utilizing the same protein sequences database as above. Orthologous gene families were identified using OrthoFinder v2.3.7 (Emms and Kelly 2019), from which 2562 one‐to‐one single‐copy orthologous genes were selected for phylogenetic inference. Sequence alignments were performed using MAFFT v7.475 (Katoh and Standley 2013), and the aligned sequences were further trimmed using Gblocks with default settings. Based on the concatenated alignment of all single‐copy orthologous genes, a maximum likelihood (ML) phylogenetic tree was built using IQtree v1.6.12 (Nguyen et al. 2015) with the best‐fit model (Q.bird+F + I + I + R7) estimated by ModelFinder (Kalyaanamoorthy et al. 2017). Branch support was assessed with 1000 ultrafast bootstrap replicates to evaluate topological robustness.

Divergence times were estimated using MCMCtree program in PAML v4.9 (Yang 2007) with the following parameters: ‐‐nsample 20,000, −‐burnin 500,000, −‐sampfreq 2000. To calibrate the molecular dating, we applied three known time points obtained from the TimeTree database (http://www.timetree.org) (Chiroptera origin: 57.9–64 million years ago (Mya)); *Rhinolophus ferrumequinum* ‐ *Rousettus aegyptiacus* split: 55.8–61.3 Mya; *Molossus molossus* ‐Vespertilionidae divergence: 38.7–48.1 Mya. To ensure the reliability of our analysis, we used Tracer v1.7.1 (Rambaut et al. 2018) to assess the convergence of the posterior distributions for all parameters. We checked the Effective Sample Size (ESS) for each parameter, ensuring that all ESS values were greater than 200.

For downstream analyses of genome size and chromosomal evolution (described in subsequent sections), we restricted the dataset to species with chromosome‐level assemblies (21 vespertilionid species and five outgroups). From these, OrthoFinder identified 19,886 orthologous gene families, including 6953 one‐to‐one single‐copy orthologs. Sequence alignment and ML tree reconstruction followed the same protocols as described above. This curated dataset provided a robust phylogenetic foundation for comparative genomic investigations.

### 2.7 Analysis of Correlation Between TE Content and Genome Size

To examine the contribution of TEs to genome size variation within the family Vespertilionidae, we performed a correlation analysis between TE content and genome size while accounting for potential phylogenetic influences. Initially, we conducted a standard correlation test using the R package *caper*. When a linear model was fitted to the data, the estimated lambda parameter was found to be 1 for overall TE content as well as for each TE type individually. A lambda near 1 indicates a strong phylogenetic signal, suggesting that evolutionary relationships among species could introduce bias into conventional correlation analyses. To address this issue and obtain unbiased estimates, we applied phylogenetic generalized least squares (PGLS) analysis, which incorporates phylogenetic information to correct for such biases and provide more robust estimates of the relationship between TE content and genome size. The PGLS analysis was implemented using the caper package in R, with the phylogeny derived from our previously reconstructed maximum likelihood tree. This method ensured that the correlations reflected true biological associations rather than artefacts of shared ancestry.

### 2.8 Gene Family Evolution

To investigate genome‐wide gene family evolution within the vespertilionid genomes, we inferred the expansion and contraction of gene families using CAFÉ v 5 (De Bie et al. 2006). The analysis was based on a set of 19,886 orthologous genes identified from chromosome‐level genome assemblies. Prior to the analysis, we removed gene families with > 100 copies in any single species to minimize potential biases caused by rapidly evolving or highly duplicated families. We applied a random birth‐death model to estimate the number of ancestral gene families and to quantify changes (expansions or contractions) along each branch of the phylogenetic tree. Significantly expanded and contracted families on individual branches were determined with a P value threshold of 0.05, as implemented in CAFÉ's likelihood ratio test framework.

### 2.9 Positive Selection Analysis

To elucidate potential associations between gene evolution and chromosomal changes in vespertilionid bats, we implemented a dual‐method approach for identifying positively selected genes (PSGs). The analysis was conducted on a set of 6953 single‐copy orthologous genes previously identified across the studied species. First, we used the branch‐site model with the CODEML program implemented in the PAML v4.9 (Yang 2007). For this analysis, we designated each of the two clades (Pipistrellini and *Myotis*) and *P. abramus* as foreground branches to test for lineage‐specific positive selection. Likelihood ratio tests (LRT) were conducted to compare the likelihoods of the null model (model = 2, NSsites = 2, fix_omega = 1, and omega = 1) and the alternative model A (model = 2, NSsites = 2, and fix_omega = 0). P values were derived from chi‐square statistics and significance was assessed with *p* < 0.05. Second, we used the aBSREL model implemented in HyPhy v 2.5.2 (Kosakovsky Pond et al. 2020) to detect PSGs along each branch, applying a significance cutoff of *p* < 0.05. To minimize false positives, only genes identified as PSGs by both methods were retained for downstream analysis.

### 2.10 Analysis of Chromosomal Evolution in the Family Vespertilionidae

To elucidate the patterns of chromosomal evolution within vespertilionid bats, we reconstructed the ancestral karyotype and assessed genomic rearrangements using a comparative approach. This analysis aimed to trace lineage‐specific changes, such as fusions and fissions, which have shaped karyotypic diversity across the family. We selected 10 vespertilionid species with chromosome‐level genome assemblies, representing a broad range of diploid number (2n): *Myotis daubentonii* 2n = 44, *Eptesicus fuscus* 2n = 50, *Pipistrellus pygmaeus* 2n = 44, *P. abramus* 2n = 26, *Nyctalus leisleri* 2n = 44, *Nyctalus aviator* 2n = 42, *Vespertilio murinus* 2n = 38, *Plecotus auritus* 2n = 32, *Aeorestes cinereus* 2n = 28, and *Antrozous pallidus* 2n = 46, as well as the outgroup of *Molossus molossus* . Species were prioritized based on distinct diploid numbers to maximize variation signals, thereby reducing computational artefacts from karyotypically similar taxa and enhancing the signal‐to‐noise ratio for detecting fusion and fission events.

The genome of *P. pygmaeus* (all autosomes and X chromosome) served as the reference and all other genomes were aligned to the reference using LASTZ v 1.04.15 (Harris 2007) with the parameters of T = 2 C = 2 H = 2000 Y = 3400 L = 6000 K = 2200. These settings optimized alignment sensitivity for large‐scale genomic comparisons. DESCHRAMBLER (Kim, Kim, et al. 2017) was then used to generate reconstructed ancestral chromosome fragments (RACFs) with a minimum syntenic region length of 500 kb and a minimum adjacency score of 0.0001.

To further investigate chromosomal rearrangements, we performed genomic synteny analysis across the 10 vespertilionid species using MCscanX (Wang et al. 2012) with default parameters. Protein sequences were compared pairwise using BLASTP (−max_target_seqs 5, −evalue 1e‐5). Based on the resulting collinearity files, syntenic blocks were identified with a minimum of four genes per block and visualized using the python package jcvi (Tang et al. 2008). This integrated approach allowed us to map evolutionary rearrangements and assess their impact on karyotype diversification.

### 2.11 Molecular Basis and Consequence of Chromosome Fusions in *P. abramus*

To characterize the types of chromosomal fusion events that occurred in *P. abramus* (see Results), we analysed centromere positions of *P. pygmaeus* (2n = 44) in *P. abramus* based on syntenic blocks between the two species. *P. pygmaeus* was chosen as the reference because it maintains the ancestral karyotype and shows a closer relationship with *P. abramus* compared to other species (see Results). Following Yin et al. (2021), we categorized fusion events as Robertsonian fusion, joining of apical centromeres from two ancestral chromosomes, tandem fusion, attachment of an ancestral chromosome's apical centromere to another ancestral chromosome's distal telomere, and end‐to‐end fusion, joining of two ancestral chromosome telomeres. Centromeric regions in *P. pygmaeus* were determined by mapping centromeric satellite sequences from *P. pipistrellus* (Barragán et al. 2003) and *P. kuhli* (Fantaccione et al. 2005) to the genome assembly with BLASTn (Altschul et al. 1990). Additionally, tandem repeat analysis was performed using Tandem Repeats Finder v4.09.1 (Benson 1999) with the parameters: trf genomes.fa 2 7 7 80 10 50,500 ‐f ‐d ‐m, and the top repeated units of each chromosome were plotted in IGV v2.4.10 (Robinson et al. 2011).

To assess the potential involvement of TEs in chromosome fusion events, we statistically evaluated whether TEs are significantly enriched at fusion sites relative to randomly selected genomic regions. Fusion sites were defined as the genomic intervals between two reconstructed ancestral chromosome fragments (RACFs) participating in fusion events. Using bedtools v2.29.1 (Quinlan 2014) in combination with genome‐wide TE annotations, we extracted TEs located within fusion sites and their flanking regions (extending 500 kb upstream and downstream of each fusion boundary). Subsequently, we conducted 1000 permutations with GAT v1.3.5 (Heger et al. 2013) to compare the observed TE base coverage in fusion and flanking regions against the expected coverage derived from randomly sampled genomic regions. Empirical *p*‐values and effect sizes (enrichment ratios) were calculated from the permutation results, and 95% confidence intervals for the enrichment ratios were generated using bootstrap resampling.

To examine the impact of chromosome fusion events on topologically associating domains (TADs) in *P. abramus* , we performed comparative TAD boundary analysis using Hi‐C data. Our approach consisted of three main steps. First, Hi‐C reads were aligned to the *P. abramus* genome assembly and processed into a normalized contact matrix using HiC‐Pro v3.1.0 (Servant et al. 2015) at 40 kb resolution. Second, we identified TAD boundaries and calculated genome‐wide insulation scores (ISs) using the hicFindTADs function in HiCExplorer v2.2.1.1 (Wolff et al. 2020). Third, we statistically compared IS values at fusion sites against genome‐wide averages to test whether fusion events preferentially occurred at TAD boundaries with reduced insulation scores.

### 2.12 Functional Enrichment Analysis

Gene Ontology (GO) enrichment analysis was performed using KOBAS v3.0 (Bu et al. 2021). Terms with a *p*‐value < 0.05 were considered statistically significant.

## 3 Results and Discussion

### 3.1 Genome Assembly and Annotation of *Pipistrellus abramus*

**FIGURE 1:**
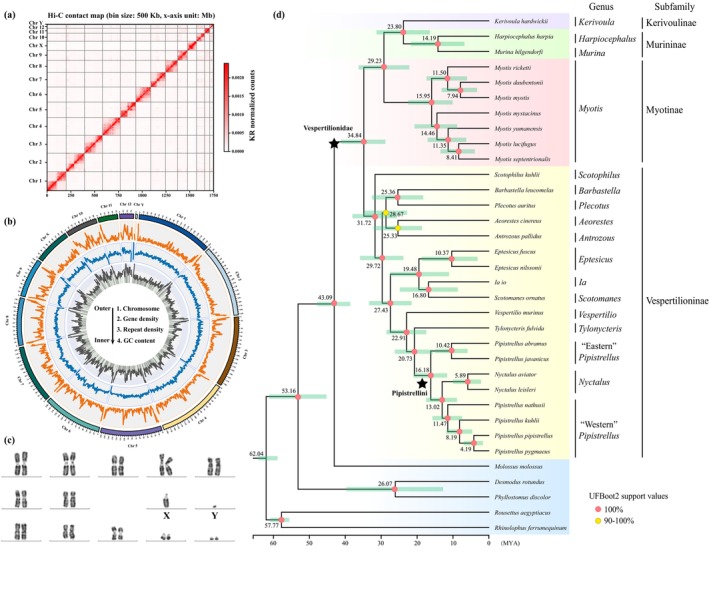
Genome assembly of *P. abramus* and phylogenetic analysis of the Vespertilionidae family. (a) Hi‐C contact heatmap illustrating inter‐chromosomal interactions. (b) Circos plot displaying genomic features arranged from the outer to inner circle: Chromosome ideograms, gene density, repeat density, and GC content (%). (c) G‐banding karyotype of *P. abramus* . (d) Phylogenetic tree reconstructed from 2562 one‐to‐one single‐copy orthologous genes with estimated divergence time among 34 bat species. MYA: Million years ago.

We generated a chromosome‐level genome assembly for *P. abramus* through integrated analysis of 165.55 Gb Nanopore long‐reads and 206.44 Gb Hi‐C reads data. *De novo* assembly of Nanopore reads yielded a 1.76 Gb draft genome (contig N50: 57.07 Mb) after redundant contigs removal, consistent with GenomeScope size estimation (Figure S2). Chromosomal scaffolding using Hi‐C data achieved 99.57% sequences anchoring to 14 pseudochromosomes (12 autosomes, X and Y chromosome) with scaffold N50 of 163.27 Mb (Figure 1a,b and Tables S5 and S6). This chromosomal configuration aligns with cytogenetic results (2n = 26, figure 1c; Lin et al. 2002; Wu et al. 2009).

To evaluate the quality of our genome assembly, we employed BUSCO, a widely recognized tool for evaluating genomic completeness. This analysis yielded a completeness metric of 95.8% (Table S6), which aligns favorably with previously reported high‐quality bat genome assemblies (Figure S3). Further supporting the assembly's integrity, high mapping rates were observed for Illumina short reads (99.85%), Nanopore long reads (99.63%), and RNA‐seq reads (95.51%), indicating both comprehensive coverage and sequence accuracy. The assembly also achieved a Consensus Quality Value (QV) of 39.2, reinforcing its high base‐level accuracy. In addition, strong syntenic conservation with chromosome‐level assemblies of two related species ( *P. pipistrellus* and *P. pygmaeus* , see below) underscored the structural reliability and high quality of our genome assembly.

Within the assembled genome, repetitive sequences spanned 661.3 Mb, accounting for 37.68% of the total genome. These repeats consisted predominantly of transposable elements (TEs, 34.8%) and tandem repeats (2.25%) (Table S7). Following repeat masking, we annotated 23,532 protein‐coding genes with an average length of 28,391.69 bp. BUSCO assessment of the annotated gene set demonstrated a completeness of 98.9%, and functional annotations were successfully assigned to the majority of these genes (21,376 genes, 90.84%) (Table S8). Together, these results affirm the high continuity, accuracy, and functional comprehensiveness of the *P. abramus* genome assembly, providing a robust foundation for downstream evolutionary analyses.

### 3.2 Phylogenetic Relationships and Divergence Time Estimates Within Vespertilionidae

To elucidate the phylogenetic relationships and divergence times within Vespertilionidae, we employed OrthoFinder to identify 2562 one‐to‐one single‐copy orthologous genes across 34 bat species, including 29 vespertilionid species and five outgroup taxa. A maximum likelihood (ML) phylogenetic tree reconstructed from these genes revealed two well‐supported clades: one encompassing three subfamilies (Kerivoulinae, Murininae and Myotinae) and the other corresponding to Vespertilioninae (Figure 1d). Additionally, our results confirmed the paraphyly of the genus *Pipistrellus* as reported in recent studies (Dool and Puechmaille 2025; Zhukova et al. 2025). Specifically, a “Western” *Pipistrellus* clade (distributed in Europe) showed closer affinity to *Nyctalus*, while an “Eastern” *Pipistrellus* clade (from Asia) occupied a more basal position relative to this group (Figure 1d). This geographic structuring underscores the complex evolutionary history of this genus.

Divergence time estimation placed the origin of Vespertilionidae at approximately 34.84 million years ago (Mya) (95% highest posterior density (HPD): 28.82–41.24 Mya). This estimate is slightly younger than that proposed by Hao et al. (2024), which reported an origin around 35.4–42.4 Mya. The discrepancy may stem from differences in calibration points used, selected molecular markers, or taxon sampling. Notably, our result aligns closely with the genome‐wide analysis conducted by Liu et al. (2024), supporting the robustness of our divergence time reference. Within Vespertilionidae, the origins of *Myotis* and Pipistrellini were estimated at 15.95 Mya (95% HPD: 10.09–22.50 Mya) and 16.18 Mya (95% HPD: 11.62–20.99 Mya), respectively (Figure 1d). These estimates provide a refined temporal framework for understanding the diversification dynamics of these ecologically diverse bat groups.

### 3.3 TEs Drive Genome Expansion in Vespertilionidae

**FIGURE 2:**
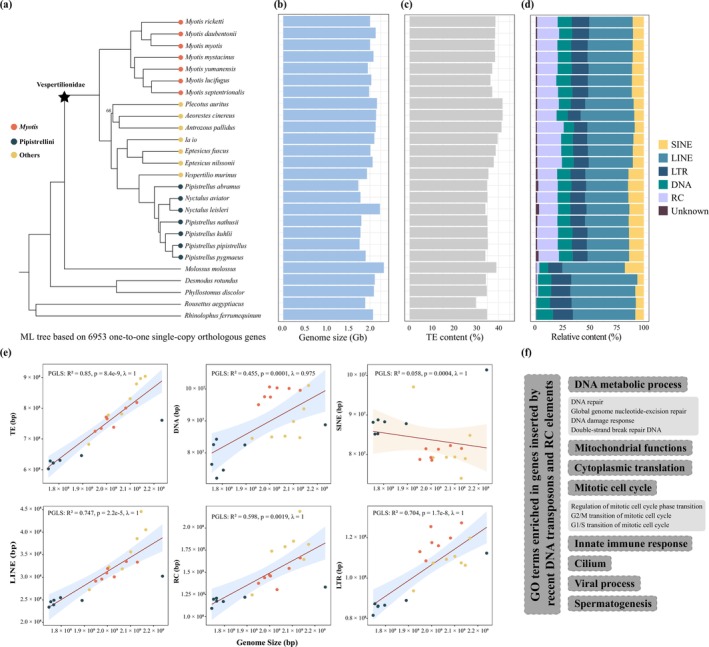
Phylogenetic relationships and genome size evolution in the family Vespertilionidae. (a) Maximum‐likelihood phylogenetic tree inferred from 6953 one‐to‐one single‐copy orthologous genes across 26 bat species. All nodes show 100% bootstrap support except one (indicated on the tree). (b) Bar plot displaying genome sizes for each of the 26 bat species. (c) Bar plot showing the proportion of total TEs in each genome. (d) Stacked bar chart illustrating the relative content of different TE types in each genome. (e) Association between genome size and the total content of TEs, as well as specific TE types, assessed using phylogenetic generalized least squares (PGLS) regression analysis. TE types include SINE (short interspersed nuclear elements), LINE (long interspersed nuclear elements), LTR (long terminal repeat elements), DNA (DNA transposons), and RC (rolling‐circle transposons). (f) Significantly enriched GO terms for genes located near insertions of RC and recent DNA transposons.

The ancestral genome size of Vespertilionidae is estimated at 1.68 Gb (Table S9), substantially smaller than that of extant species (ranging from 1.76 Gb in *P. abramus* to 2.23 Gb in *Nyctalus leisleri* ; Figure 2a,b and Table S10). To assess the contribution of TEs to this genome expansion, we performed a comparative repeat analysis across 21 vespertilionid and five non‐vespertilionid bat species. For consistency, repeat sequences of all species were annotated using the same custom repeat library developed for *P. abramus* .

Our analysis revealed that vespertilionid bats exhibit conserved repeat sequence proportions, with TE‐derived sequences accounting for 34.08% to 41.85% of their genomes (Table S11). Notably, vespertilionids exhibited a trend toward higher TE content compared to non‐vespertilionid bats (vespertilionids: 37.34% *n* = 21; non‐vespertilionids: 34.54% *n* = 5), although this difference was not statistically significant (*p* = 0.068, Mann–Whitney *U* test). This pattern suggests that lineage‐specific TE accumulation may drive genome size expansion within this family. Consistent with patterns observed in other mammals (Platt et al. 2018), long interspersed nuclear elements (LINEs) constituted the most abundant TE class in vespertilionid genomes, ranging from 13.14% to 22.65% of the total genome sequence. rolling‐circle (RC) elements represented the second most prevalent category (5.97%–10.29%), followed by LTR retrotransposons (4.69%–6.28%), SINEs (3.78%–5.18%), and DNA transposons (3.98%–5.04%) (Figure 2a,c,d; Table S11).

To evaluate the relationship between TE content and genome size variation, we compared the relative amount of TEs with genome size differences. Phylogenetically corrected Pearson correlation analysis demonstrated a strong positive correlation between total TE content and genome size among vespertilionids (*R* ^2^ = 0.85, *p* = 8.4e‐9; Figure 2e), aligning with previous studies (Chalopin et al. 2015; Canapa et al. 2016; Osmanski et al. 2023). Further analysis of individual TE types revealed significant positive correlations for LINEs (*R* ^2^ = 0.747, *p* = 2.2e‐5), LTR elements (*R* ^2^ = 0.704, *p* = 1.7e‐8), DNA transposons (*R* ^2^ = 0.455, *p* = 0.0001), and RC elements (*R* ^2^ = 0.455, *p* = 0.0019). In contrast, SINEs exhibited a significant negative correlation (*R* ^2^ = 0.058, *p* = 0.0004; Figure 2e). This inverse relationship may reflect evolutionary trade‐offs or distinct regulatory mechanisms governing SINE activity.

Consistent with previous findings (Pritham and Feschotte 2007; Paulat et al. 2023), we detected a specific expansion of RC elements in vespertilionid bats, characterized by 6%–11% divergence from consensus sequences (Figure S4). Vespertilionids are the only mammals known to harbour RC elements invasions, a phenomenon first reported in *Myotis* (Pritham and Feschotte 2007) and later confirmed in over 200 high‐quality mammalian genomes (Paulat et al. 2023; Osmanski et al. 2023). Additionally, recent accumulations of DNA transposons (< 4% divergence from consensus sequences) we observed across all vespertilionid bats (Figure S5). These patterns have been attributed to horizontal transfer (HT) events (Paulat et al. 2023), which are known to drive genome size expansion in other taxa, such as mosquitoes (Melo and Wallau 2020) and other insects (Peccoud et al. 2017). The high HT frequency in vespertilionids may facilitate rapid diversification within the family (Paulat et al. 2023), possibly through TE‐mediated genomic innovations (Oliver and Greene 2012).

To explore why vespertilionids serve as HT hotspots, we examined insertion sites of these specific RC elements and recent DNA transposons in 21 high‐quality vespertilionid genomes. Our analysis revealed that RC elements were inserted in exons of 7–263 genes and in promoters of 107–531 genes (Table S12). Similarly, recent DNA transposons were found in exons of 6–375 genes and in promoters of 43–988 genes (Table S13). Functional enrichment analysis of genes associated with these TEs demonstrated significant enrichment in Gene Ontology (GO) terms related to mitochondrial functions, cytoplasmic translation, DNA metabolic process (e.g., DNA repair, global genome nucleotide‐excision repair, DNA damage response and double‐strand break repair), mitotic cell cycle, viral process, innate immune response, and spermatogenesis (Figure 2f; Figures S6–S9 and Tables S14–S17). The enrichment of RC and recent DNA transposons in genes involved in DNA metabolism suggests a potential mechanistic basis for TE tolerance in Vespertilionidae: insertions may yield modified protein variants that enhance the efficiency of TE excision and integration, thereby mitigating deleterious effects. For example, one such gene, *HINFP*, has been shown to maintain genome stability by repressing TEs (Nirala et al. 2021), indicating a possible co‐evolutionary relationship between host genomic safeguards and TE activity. This pattern highlights how vespertilionids may have evolved mechanisms to accommodate TE proliferation, potentially facilitating their role as HT hotspots.

### 3.4 Chromosomal Evolution in Vespertilionidae

**FIGURE 3:**
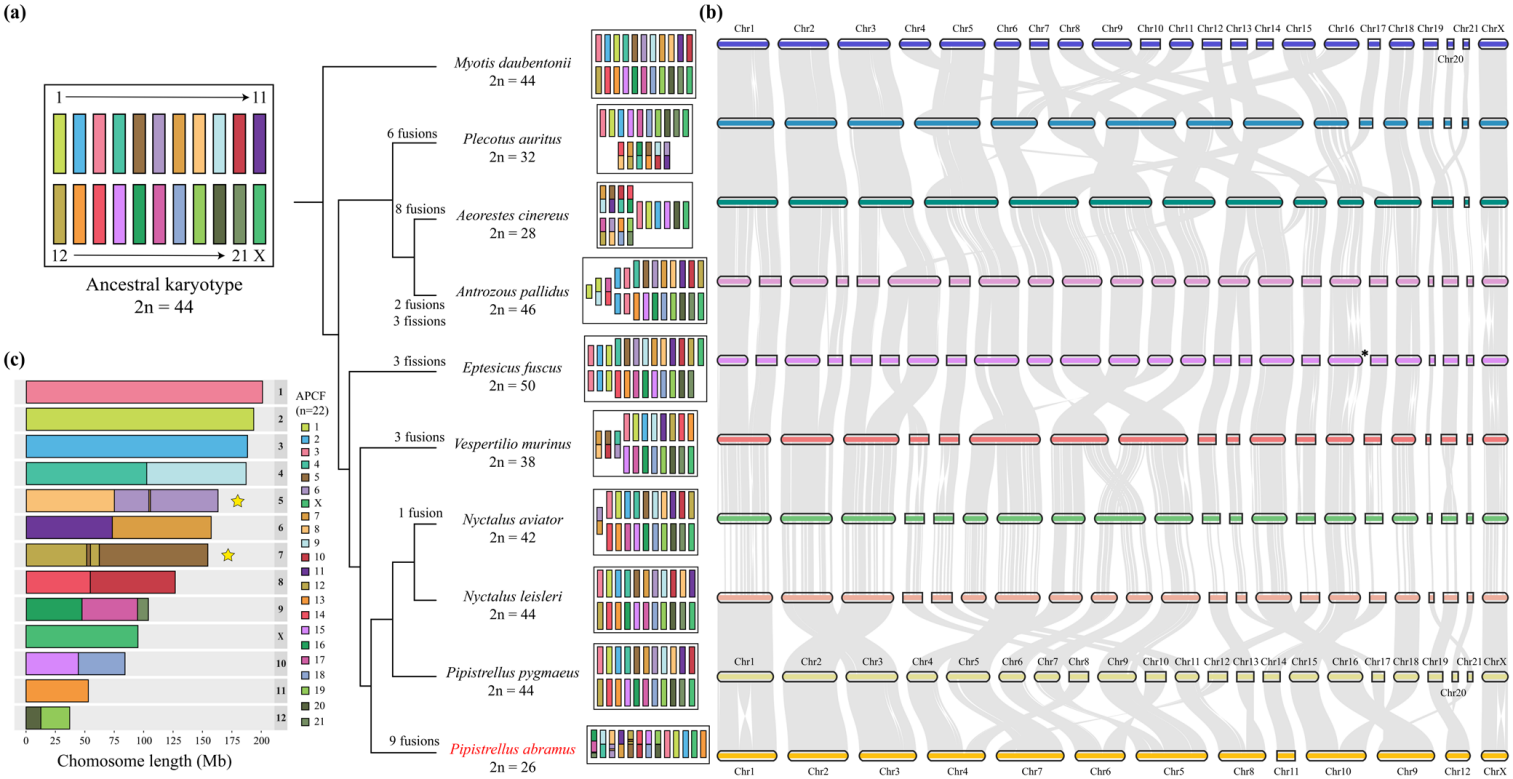
Chromosomal evolution in the family Vespertilionidae. (a) Reconstruction of ancestral chromosomes in Vespertilionidae with specific chromosomal changes (fusions and fissions) highlighted along branches from ancestral karyotype to each extant species. (b) Whole‐genome synteny across 10 vespertilionid species. The asterisk (*) marks the acrocentric chromosome in *Eptesicus fuscus* that corresponds to the small bi‐armed chromosome 15 in *P. pygmaeus* . (c) Ideogram of the reconstructed karyotype for *P. abramus* aligned with ancestral predicted chromosome fragments (APCFs). The star (★) indicates a chromosome that underwent an inversion following a chromosomal fusion event.

To elucidate patterns of chromosomal evolution within Vespertilionidae, we reconstructed the ancestral karyotype using chromosome‐level genome assemblies from 10 vespertilionid species and one outgroup via DESCHRAMBLER. This analysis identified 177 conserved genomic segments (average size: 10.39 Mb; range: 0.31–75.91 Mb), which facilitated the reconstruction of 22 ancestral chromosomes—comprising 21 autosomes and one X chromosome (2n = 44) (Figure 3a and Table S9). These results align with previous molecular cytogenetic studies (e.g., Bickham 1979b; Zima 1982). Validation using DESCHRAMBLER with five additional outgroups confirmed the robustness of this ancestral karyotype reconstruction (Table S9).

To trace lineage‐specific chromosomal changes, we compared the ancestral karyotype with those of extant vespertilionid species. Our analysis revealed that seven *Myotis* and five Pipistrellini species have retained the ancestral karyotype, characterized by three large bi‐armed autosomes, one small bi‐armed autosome, and 17 acrocentric autosomes—consistent with earlier cytogenetic reports (Bickham 1979a; Ao et al. 2006). In contrast, other species exhibited derived karyotypes shaped primarily by chromosomal fusions and fissions (Figure 3a). Specifically, we identified eight fusion events leading to a reduced diploid number in *Aeorestes cinereus* (2n = 28), six fusions in *Plecotus auritus* (2n = 32), three fusions in *Vespertilio murinus* (2n = 38), and one fusion in *Nyctalus aviator* (2n = 42). These findings align with previous cytogenetic studies (Kulemzina et al. 2011 for *Plecotus auritus* and *Vespertilio murinus* ; Harada et al. 1982 for *Nyctalus aviator* ). In *Eptesicus fuscus* (2n = 50), the karyotype originated from three fission events involving the three large ancestral bi‐armed chromosomes, accompanied by three successive inversions that converted the small ancestral bi‐armed chromosome into an acrocentric chromosome (Figure 3b and Figure S10, see also Bickham (1979b)). Similarly, in *Antrozous pallidus* (2n = 46), we detected three fission events that occurred in the large bi‐armed ancestral chromosomes, followed by two independent fusion events, corroborating earlier cytogenetic findings (Bickham 1979b). In *P. abramus* , karyotype evolution involved two fusions of three ancestral chromosomes forming chromosome 9 (chr9), along with seven additional independent fusions that gave rise to chromosomes 4–8, 10, and 12 (chr4–8, 10, 12). The absence of shared fusion events across these lineages underscores the independent and lineage‐specific nature of chromosomal evolution in vespertilionids.

Genomic synteny analysis further confirmed that chromosomal fusion and fission represent the primary mechanism driving karyotypic diversity among vespertilionid bats (Figure 3b). These results provide support for the concept of karyotypic orthoselection (White 1975), wherein certain types of structural rearrangements are evolutionarily favoured within a lineage, as previously proposed by Baker et al. (1985). The recurrence of specific rearrangement types underscores the role of genomic architecture and selective pressures in shaping chromosomal evolution among vespertilionid bats, contributing to their diversification and adaptation.

### 3.5 Molecular Mechanisms Underlying Divergent Karyotype Evolution in Pipistrellini Versus *Myotis*

Striking contrasts in karyotype evolution characterize these two bat lineages. While *Myotis* bats uniformly retained the ancestral karyotype (2n = 44, Bickham et al. 2004), the tribe Pipistrellini exhibits extensive chromosomal reorganization, with 2n ranging from 26 to 44 (Pathak and Sharma 1969; Volleth et al. 2001, see Introduction). To investigate whether specific genetic adaptations underlie this striking contrast—specifically, the propensity for extensive karyotype reorganization in Pipistrellini versus the remarkable karyotype stasis in *Myotis*—we performed genome‐wide scans for positively selected genes (PSGs). Using complementary methods (PAML and HyPhy), we identified PSGs in *P. abramus* (representing the variable Pipistrellini lineage) alongside representative taxa from both lineages for comparative analysis (Table S18). Notably, PSGs in *P. abramus* were significantly enriched for GO terms associated with chromosomal integrity and dynamics, including chromatin binding, chromatin remodelling, DNA repair, cellular response to DNA damage stimulus, and cell cycle (e.g., mitotic centrosome separation and G2/M transition of mitotic cell cycle) (Table S19). Similarly, PSGs across Pipistrellini showed enrichment for cell cycle‐related terms, such as positive regulation of G1/S transition of mitotic cell cycle (Table S20). The proteins encoded by these PSGs are critical for maintaining chromosome integrity and genome stability. Their positive selection suggests an adaptive mechanism that have facilitated the tolerance or propagation of large‐scale karyotype changes in Pipistrellini, particularly in *P. abramus* . Conversely, PSGs in *Myotis* revealed no enrichment for chromosome‐associated GO terms (Table S21), aligning with the observed karyotypic stasis in this genus. This absence of selection pressure on chromosomal maintenance machinery may reflect evolutionary constraints preserving the ancestral karyotype configuration. Nevertheless, an alternative interpretation warrants consideration: The observed selection signals in Pipistrellini could represent lineage‐specific adaptations unrelated to chromosomal restructuring—acting neither as drivers of fusion/fission events nor as stabilizers of resultant karyotypes. Such adaptations might instead reflect other ecological or physiological specializations. Future functional validation of these PSGs will be essential to resolve their precise role in karyotype evolution.

**FIGURE 4:**
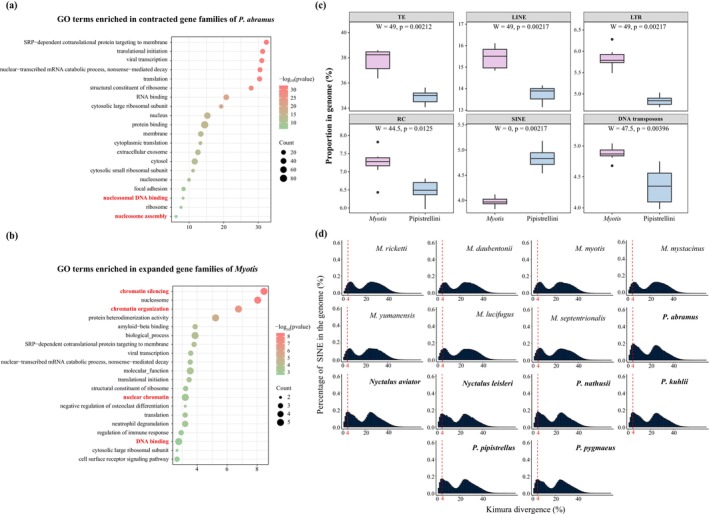
Molecular basis underlying extensive karyotype changes in Pipistrellini versus conserved karyotype in *Myotis*. (a) Top 20 significantly enriched GO terms associated with genes from contracted gene families in *P. abramus* . (b) Top 20 significantly enriched GO terms associated with genes from expanded gene families in *Myotis*. (c) Comparison of the proportion of each TE type in genomes of Pipistrellini versus *Myotis*. (d) Genomic landscape of recent SINE insertions (defined as ≤ 4% sequence divergence from consensus sequences) across species in Pipistrellini and *Myotis*.

To investigate whether the evolution of gene families is associated with karyotypic changes in Pipistrellini, we conducted a gene family analysis in a phylogenetic tree of 26 bat species using the software Café. Our analysis identified significantly expanded and contracted gene families along the branches leading to *Myotis*, Pipistrellini and *P. abramus* (Figure S11). Functional enrichment analysis revealed that genes within the contracted gene families in *P. abramus* showed significant enrichment for Gene Ontology (GO) terms associated with chromosome integrity and genome stability (18 out of 205 enriched terms, Table S22). These included nucleosome assembly (GO:0006334), negative regulation of chromatin silencing (GO:0031936), chromosome condensation (GO:0030261), chromatin silencing (GO:0006342), negative regulation of DNA recombination (GO:0045910), negative regulation of telomere maintenance via telomerase (GO:0032211), and positive regulation of cell cycle arrest (GO:0071158) (Figure 4a and Table S22). In contrast, no enrichment for comparable GO terms related to genome stability was observed for genes within the expanded gene families in Pipistrellini or *P. abramus* , nor within the contracted gene families in Pipistrellini (Tables S22 and S23). This specific pattern of contraction in *P. abramus* suggests that the loss of these gene families may compromise genome stability mechanisms. We propose that this genomic instability could be a significant contributing factor to the extensive karyotypic changes observed in *P. abramus* , consistent with similar mechanisms reported in parrots (Huang et al. 2022) and clitellates (Vargas‐Chávez et al. 2025). Supporting this hypothesis, genes within expanded gene families in *Myotis* were enriched for GO terms functionally analogous to those contracted in *P. abramus* . These included chromatin silencing (GO:0006342), nucleosome (GO:0000786), and chromatin organization (GO:0006325), involving genes such as *HIST1H2AD*, *H2AFJ*, *HIST2H2AB*, *HIST2H2AC*, and *HIST1H2AK* (Figure 4b and Table S24). The expansion of these particular gene families may underpin the high degree of karyotype conservation characteristic of the *Myotis* lineage.

To investigate the potential association between TEs and karyotypic changes (Fedoroff 2012; Cornet et al. 2024) due to their sequence similarity and repetitive nature across chromosomes (Hedges and Deininger 2007), we compared the TE landscapes of Pipistrellini and *Myotis* bats. Our analysis revealed that the overall TE content was significantly higher in *Myotis* than in Pipistrellini (*p* = 0.000212, W = 49, Wilcoxon rank sum exact test, Figure 4c). When examining specific TE types, Pipistrellini exhibited a higher amount of SINEs but lower amounts of other TE types compared to *Myotis* (*p* < 0.05 in all Wilcoxon rank sum exact tests, Figure 4c). Notably, a recent expansion of SINEs—characterized by less than 4% divergence from consensus sequences—was observed in Pipistrellini relative to *Myotis* bats (Table S25 and Figure 4d). This suggests that SINEs may play a critical role in the genome evolution and adaptation of Pipistrellini, as they can induce structural variants and modulate gene expression (Schmitz 2012).

Further analysis of insertion sites of these recent SINEs in seven Pipistrellini genomes showed that 74 genes had insertions in exons and 1411 genes in promoters (Table S26). Functional enrichment analysis indicated that these genes were significantly associated with GO terms related to genome stability. For instance, among genes with SINE insertions in exons, 5 out of 55 enriched terms were linked to stability mechanisms, while for promoter insertions, 20 out of 338 terms were relevant (Table S27). Key enriched processes included double‐strand break repair via nonhomologous end joining (e.g., involving genes like *RNF168*, *KDM2A* and *BABAM1*), DNA damage response (e.g., *PCNA*, *RPA2*, and *RPS3*), nuclear chromosome organization (e.g., *TINF2*, *ORC5*, *TFIP11*, and *TERF2*), and DNA repair (e.g., *RAD51C* and *MSH3*) (Figures S12 and S13 and Table S27). These findings imply that the recent expansion of SINEs could contribute to the extensive karyotypic changes in Pipistrellini by disrupting genes central to genomic integrity, supporting the important roles of TE insertions in chromosomal diversity (Klein and O'Neill 2018). Overall, our integrated analyses highlight the interplay between positive selection, gene family dynamics, and TE activity in shaping karyotype evolution. The divergent evolutionary trajectories of Pipistrellini and *Myotis* underscore the complexity of genomic adaptations driving chromosomal diversity in vespertilionid bats.

### 3.6 Molecular Basis and Consequence of Chromosome Fusions in *P. abramus*

**FIGURE 5:**
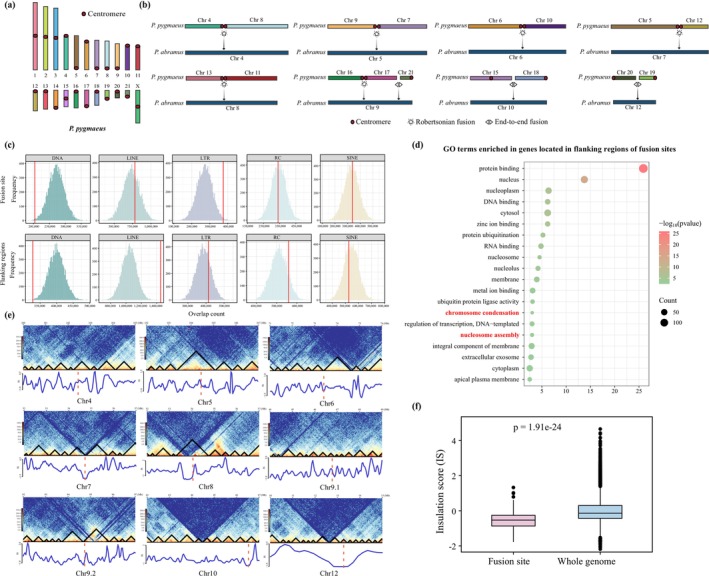
Molecular mechanism and consequence of chromosomal fusions in *P. abramus* . (a) Karyotype of *P. pygmaeus* with annotated centromere positions. (b) Classification of chromosomal fusion types in *P. abramus* identified by comparing with the karyotype of *P. pygmaeus* . (c) Enrichment analysis of each TE type at chromosomal fusion sites and adjacent flanking regions. Histograms illustrate the distribution of TE counts from 1000 randmized control regions matched in size to the fusion sites. The red line represents the observed TE count for each type within fusion regions. (d) Top 20 significantly enriched GO terms for genes located in flanking regions of the chromosome fusion sites. Terms related to the processes involved in chromosomal biology are shown in red. (e) Topologically associating domains (TADs) identified at fusion site *P. abramus* chromosomes, with corresponding insulation score (IS) shown as dashed lines. (f) Comparative analysis of insulation scores (ISs) at fusion sites versus the genome‐wide average (Wilcoxon test, *p* = 1.91e‐24).

As described above, the karyotype of *P. abramus* (2n = 26) has evolved from the ancestral vespertilionid karyotype (2n = 44) through nine chromosomal fusion events. To elucidate the molecular mechanisms underlying these rearrangements, we integrated centromeric satellite sequence data from two related *Pipistrellus* species ( *P. pipistrellus* and *P. kuhli* ) with tandem repeat analysis, enabling confident identification of all centromere positions in the *P. pygmaeus* genome assembly (Figure 5a). Comparative centromere positioning analysis with *P. pygmaeus* revealed that chromosome 9 (chr9) of *P. abramus* originated through one Robertsonian (Rb) fusion and one end‐to‐end fusion, while chromosomes 4–8 (chr4‐8) were formed by Rb fusions, and chromosomes 10 and 12 arose via end‐to‐end fusions (Figure 5b). Notably, inversions were observed subsequent to two of the fusion events in chr5 and chr7 (Figure 3c). These irreversible fusion‐with‐inversion events are relatively rare in animals, resembling the “fusion‐with‐mixing” patterns previously reported in chromosome evolution of metazoans (Simakov et al. 2022), hemichordates (Lin et al. 2024) and octopus (Yoshida et al. 2025), suggesting a conserved yet specialized mechanism of chromosomal evolution.

To investigate the potential role of TEs in these fusion events, we examined whether TEs are significantly enriched at fusion sites and/or their flanking regions across eight *P. abramus* chromosomes (chr4‐10 and chr12). These nine fusion sites, defined as the interval between two reconstructed ancestral chromosome fragments (RACFs) involved in fusion events, accounted for 6.02 Mb with an average length of 668.38 kb, ranging from 6.42 kb to 3.43 Mb (Table S28). Permutation tests revealed significant enrichment of LTR sequences at fusion sites (*p* = 0.011) and LINE sequences in flanking regions (*p* = 0.001) relative to random expectations (Figure 5c). These findings suggest a potential association between TEs and chromosomal fusion events in *P. abramus* , consistent with observations in wood white butterflies (Höök et al. 2023). However, it is important to consider that fusion sites may represent relics of telomere ends, which are characterized by highly repetitive sequences. Telomeres typically contain tandem repeats of short DNA sequences, and these regions can also harbour various types of transposable elements (Pardue and DeBaryshe 2003; Casacuberta 2017; Lu and Liu 2024). Therefore, the observed enrichment of LTRs at fusion sites may, in part, reflect the inherent repetitiveness of telomeric regions rather than a specific role of LTRs in the fusion process. Overall, our current results suggest a potential correlation between the presence of TEs and the occurrence of fusion events during the process of chromosomal evolution. While TEs may accumulate following fusion or be present due to the nature of highly repetitive chromosome ends, a direct causal role of TEs in driving fusions cannot be confirmed based on the data presented in this manuscript. The potential role of TEs in chromosomal rearrangements, including fusions, has been a topic of interest in the literature (Lönnig and Saedler 2002; Ahola et al. 2014; Hofstatter et al. 2022; Höök et al. 2023; Cornet et al. 2024). Studies have shown that TEs can contribute to genomic instability and structural variations (Kidwell and Holyoake 2001; Balachandran et al. 2022), but the specific mechanisms by which they drive or facilitate fusions remain to be fully elucidated. Our findings add to this ongoing discussion and highlight the need for further investigation into the functional roles of TEs in chromosomal evolution.

We next identified 103 protein‐coding genes located at chromosomal fusion sites (Table S29). Functional enrichment analysis demonstrated that these genes are significantly enriched in GO terms associated with neuronal function, including synapse organization, neurotransmitter receptor activity, chemical synaptic transmission, and nervous system process (Table S30). This suggests a potential link between neural development and genomic structural changes, though the exact functional implications warrant further investigation. Among these genes, *SYCP2* is particularly noteworthy, as it has been shown to promote DNA double‐strand break repair via transcription‐coupled homologous recombination (Wang et al. 2024), highlighting a possible role in stabilizing fusion‐derived genomic alterations.

To contextualize the genomic environment of fusion sites, we annotated regions spanning 500 kb upstream and downstream of each fusion site, identifying 221 proximal genes (Table S31). These genes showed significant enrichment in GO terms related to chromosome biology (e.g., chromosome condensation, chromatin DNA binding, negative regulation of chromatin silencing, and negative regulation of DNA recombination) (Figure 5d and Table S32). The clustering of chromosomal biology‐related genes near fusion regions implies their potential involvement in the mechanisms driving fusion events. Several of these genes have been shown to play critical roles in maintaining genome integrity and stability. For example, *PARP1* plays multifaceted roles in DNA repair and chromatic remodelling (Ray Chaudhuri and Nussenzweig 2017), while *RECQL4* is essential for DNA double‐strand break repair and the maintenance of telomeres (Croteau et al. 2012). Notably, multiple genes belong to the linker histone family (e.g., *HIST1H1E*, *HIST1H1A* and *HIST1H1B*) which regulate chromatin structure and accessibility. Additionally, four genes (*FANCA*, *LARP7*, *CTHRC1*, and *SAP130*) were identified as PSGs in *P. abramus* . Among these, *FANCA* has been shown to play important roles in genome maintenance by promoting DNA double‐strand break repair (Benitez et al. 2018) and *LARP7* acts as a key regulator of telomerase assembly (Collopy et al. 2018). The prevalence of genome stability‐related genes near fusion sites suggests that protein‐coding genes might play important roles in the formation of fusion events. These genes could be directly involved in the mechanisms that lead to chromosomal fusions, or they may be by‐products of the fusion events, subsequently contributing to the maintenance of genome stability. Future work employing functional assays, such as CRISPR‐based perturbations, will be essential to validate these genes' contributions and unravel the causal pathways underlying karyotype diversification.

Finally, to assess the functional impact of extensive chromosome fusions, we evaluated their effects on topologically associating domains (TADs). Our analysis revealed that most fusion sites were located at TAD boundaries (Figure 5e) and exhibited significantly lower insulation scores (ISs) compared to the genome‐wide average (fusion sites: *n* = 161, whole‐genome: *n* = 25,350, Wilcoxon test, *p* = 1.91e‐24, Figure 5f). This indicates that chromosomal fusions in *P. abramus* may have limited disruptive effects on higher‐order chromatin architecture, thereby preserving genomic organization and potentially facilitating adaptation—a phenomenon consistent with observations in muntjac deer (Yin et al. 2021). To further evaluate whether these chromosomal rearrangements influence transcriptional activity, we propose a comparative transcriptomic analysis between *P. abramus* and related *Pipistrellus* species with no fusion events. Such an approach would clarify whether genes located within or near the fusion sites exhibit altered expression profiles compared to unfused genomic regions (e.g., Li et al. 2023), elucidating the functional relevance of fusion‐mediated restructuring in adaptive evolution of *P. abramus* .

## 4 Conclusion

In this study, we present a high‐quality chromosome‐level genome assembly for *Pipistrellus abramus* , a vespertilionid bat species characterized by a relatively low diploid number (2n = 26) within Chiroptera. By integrating multiple high‐quality genome assemblies from other vespertilionid bats, we reconstructed an updated and robust phylogeny of the family Vespertilionidae, providing a comprehensive framework for evolutionary analyses. Furthermore, we systematically examined the impacts of specific rolling‐circle (RC) transposons and recent DNA transposons on genomic architecture, revealing that genes targeted by these elements are functionally enriched in DNA metabolism processes. This finding suggests a potential mechanistic basis for TE tolerance in Vespertilionidae. Our reconstruction supports an ancestral karyotype of 2n = 44 for this family, with chromosomal fusions and fissions identified as the primary driver of karyotypic diversification. A notable example is an unusually derived chromosome in *P. abramus* , originating from three ancestral chromosomes through one Robertsonian fusion and one end‐to‐end fusion event. While TEs exhibit significant enrichment at fusion sites, our results underscore the involvement of genome stability‐related genes in adaptive mechanisms that either tolerate or exploit structural genomic changes. Additionally, we propose that contraction of gene families associated with genome stability may serve as an alternative mechanism facilitating extensive karyotypic evolution in mammals. Collectively, these insights advance our understanding of chromosomal dynamics and their role in speciation and adaptation, highlighting the utility of comparative genomics in elucidating evolutionary processes.

## Author Contributions

X.M. conceived and supervised the project; L.L., X.Z. and J.X. performed data analysis with the help from X.L. and X.H.; W.N., J.W. and W.S. did cytogenetic work; X.M. wrote the manuscript with the input from L.L., X.Z. and J.X.; G.H. obtained funding and edited the manuscript; F.Y. contributed valuable comments and edited the manuscript. All authors reviewed and approved the final version of the manuscript.

## Funding

This work was supported by Shanghai Science and Technology Committee Project, No. 18DZ2293800. The Shanghai Wildlife‐borne Infectious Disease Monitoring Program.

## Conflicts of Interest

The authors declare no conflicts of interest.

## Supporting information


**Figure S1:** Mitochondrial genome of *P. abramus* .
**Figure S2:** Genomescope profile of *P. abramus* based on Illumina short‐read data.
**Figure S3:** Comparative assessment of genome assembly quality in nine bat species based on BUSCO completeness scores and N50 statistics for Contigs and Scaffolds.
**Figure S4:** Sequence divergence of rolling‐circle (RC) transposons from consensus sequences across genomes of 21 vespertilionid bat species.
**Figure S5:** Sequence divergence of DNA transposons from consensus sequences across genomes of 21 vespertilionid bat species.
**Figure S6:** Top 20 significantly enriched GO terms for genes containing RC transposons insertion within their promoter regions in vespertilionid bats.
**Figure S7:** Top 20 significantly enriched GO terms for genes containing RC transposons insertion within their exon regions in vespertilionid bats.
**Figure S8:** Top 20 significantly enriched GO terms for genes containing insertion of DNA transposons in their promoter regions in vespertilionid bats.
**Figure S9:** Top 20 significantly enriched GO terms for genes containing insertion of DNA transposons in their the exon regions in vespertilionid bats.
**Figure S10:** Comparative alignment of the small bi‐armed chromosome 15 in *P. pygmaeus* with the homologous acrocentric chromosome in *Eptesicus fuscus* .
**Figure S11:** The phylogenetic tree displays counts significantly expanded (red numbers) and contracted (blue numbers) gene families identified for each bat species and evolutionary branch.
**Figure S12:** Top 20 significantly enriched GO terms for genes containing insertions of recent SINEs within their promoter regions in *Pipistrellus*.
**Figure S13:** Top 20 significantly enriched GO terms for genes containing insertions of recent SINEs within their exton regions in *Pipistrellus*.

**Table S1:** Summary of genome sequencing and transcriptomic data for *P. abramus* .
**Table S2:** Information of genome sequences for species used in homology‐based gene prediction.
**Table S3:** Detailed information about the mitogenome annotation for *P. abramus* .
**Table S4:** Information about genome sequences for 33 species used in phylogenetic reconstruction. Species with available genome annotations were shown in bold.
**Table S5:** Information of chromosome‐level genome assembly for *P. abramus* .
**Table S6:** Information of the genome assembly and quality assessment for *P. abramus* .
**Table S7:** Repeat annotation in genome assembly of *P. abramus* .
**Table S8:** Information of functional annotation and assessment of gene prediction using BUSCOs in *P. abramus* .
**Table S9:** Reconstructed lengths of ancestral chromosomes (Chr) in the family Vespertilionidae using DESCHRAMBLER. Panels (a) and (b) present chromosome lengths estimates based on reconstructions using one outgroup and five outgroups, respectively.
**Table S10:** Genome sizes of 21 vespertilionid species with chromosome‐level genome assemblies.
**Table S11:** Annotation summary of TEs annotation in 26 bat species.
**Table S12:** Number of genes containing insertions of RC transposons within promoters and exons across 21 vespertilionid species.
**Table S13:** Number of genes containing insertions of DNA transposons within promoters and exons across 21 vespertilionid species.
**Table S25:** Detailed information about the total SINE and recent SINEs (≤ 4% divergence) in vespertilionid bats.
**Table S28:** Length of chromosome fusion sites in chromosomes of *P. abramus* . Rb: Robertsonian fusion.

**Table S14:** Significantly enriched GO terms for genes containing insertions of RC transposons within their promoter regions in vespertilionid bats.
**Table S15:** Significantly enriched GO terms for genes containing insertions of RC transposons within their exon regions in vespertilionid bats.
**Table S16:** Significantly enriched GO terms for genes containing insertions of DNA transposons within their promoter regions in vespertilionid bats.
**Table S17:** Significantly enriched GO terms for genes containing insertions of DNA transposons within their exon regions in vespertilionid bats.
**Table S18:** Detailed information about the positively selected genes (PSGs) identified in *Myotis*, Pipistrellini and *P. abramus* .
**Table S19:** Significantly enriched GO terms for PSGs identified in *P. abramus* .
**Table S20:** Significantly enriched GO terms for PSGs identified in Pipistrellini.
**Table S21:** Significantly enriched GO terms for PSGs identified in *Myotis*.
**Table S22:** Significantly enriched GO terms for genes from expanded/contracted gene families in *P. abramus* .
**Table S23:** Significantly enriched GO terms for genes from expanded/contracted gene families in Pipistrellini.
**Table S24:** Significantly enriched GO terms for genes from expanded/contracted gene families in *Myotis*.
**Table S26:** Genes containing insertions of recent SINEs (≤ 4% divergence) within promoter regions and exon regions in Pipistrellini.
**Table S27:** Significantly enriched GO terms for genes containing insertions of recent SINEs within exon/promoter regions in Pipistrellini.
**Table S29:** Annotated genes located in fusion sites of *P. abramus* chromosomes.
**Table S30:** Significantly enriched GO terms for genes located in fusion sites of *P. abramus* chromosomes.
**Table S31:** Annotated genes located in flanking regions of fusion sites of *P. abramus* chromosomes.
**Table S32:** Significantly enriched GO terms for genes located in flanking regions of fusion sites of *P. abramus* chromosomes.

## Data Availability

All newly generated data in this study are available in the Sequence Read Archive (SRA) database under BioProject accession number PRJNA1173466. The genome assembly and annotation of *Pipistrellus abramus* have been deposited in the Figshare repository (https://doi.org/10.6084/m9.figshare.27684225).
