# Supplementary material for: Comparative Genomics Provide Insights Into Karyotype Evolution in Vespertilionid Bats (Vespertilionidae, Chiroptera)
Source: Mol Ecol Resour. 2026 Mar 20;26(3):e70129. doi: 10.1111/1755-0998.70129 (PMC13003292; doi:10.1111/1755-0998.70129)
Supplement: Supplementary file 1 — Figure S1: Mitochondrial genome of P. abramus . Figure S2: Genomescope profile of P. abramus based on Illumina short‐read data. Figure S3: Comparative assessment of genome assembly quality in nine bat species based on BUSCO completeness scores and N50 statistics for Contigs and Scaffolds. Figure S4: Sequence divergence of rolling‐circle (RC) transposons from consensus sequences across genomes of 21 vespertilionid bat species. Figure S5: Sequence divergence of DNA transposons from consensus sequences across genomes of 21 vespertilionid bat species. Figure S6: Top 20 significantly enriched GO terms for genes containing RC transposons insertion within their promoter regions in vespertilionid bats. Figure S7: Top 20 significantly enriched GO terms for genes containing RC transposons insertion within their exon regions in vespertilionid bats. Figure S8: Top 20 significantly enriched GO terms for genes containing insertion of DNA transposons in their promoter regions in vespertilionid bats. Figure S9: Top 20 significantly enriched GO terms for genes containing insertion of DNA transposons in their the exon regions in vespertilionid bats. Figure S10: Comparative alignment of the small bi‐armed chromosome 15 in P. pygmaeus with the homologous acrocentric chromosome in Eptesicus fuscus . Figure S11: The phylogenetic tree displays counts significantly expanded (red numbers) and contracted (blue numbers) gene families identified for each bat species and evolutionary branch. Figure S12: Top 20 significantly enriched GO terms for genes containing insertions of recent SINEs within their promoter regions in Pipistrellus. Figure S13: Top 20 significantly enriched GO terms for genes containing insertions of recent SINEs within their exton regions in Pipistrellus. [file MEN-26-e70129-s002.docx]

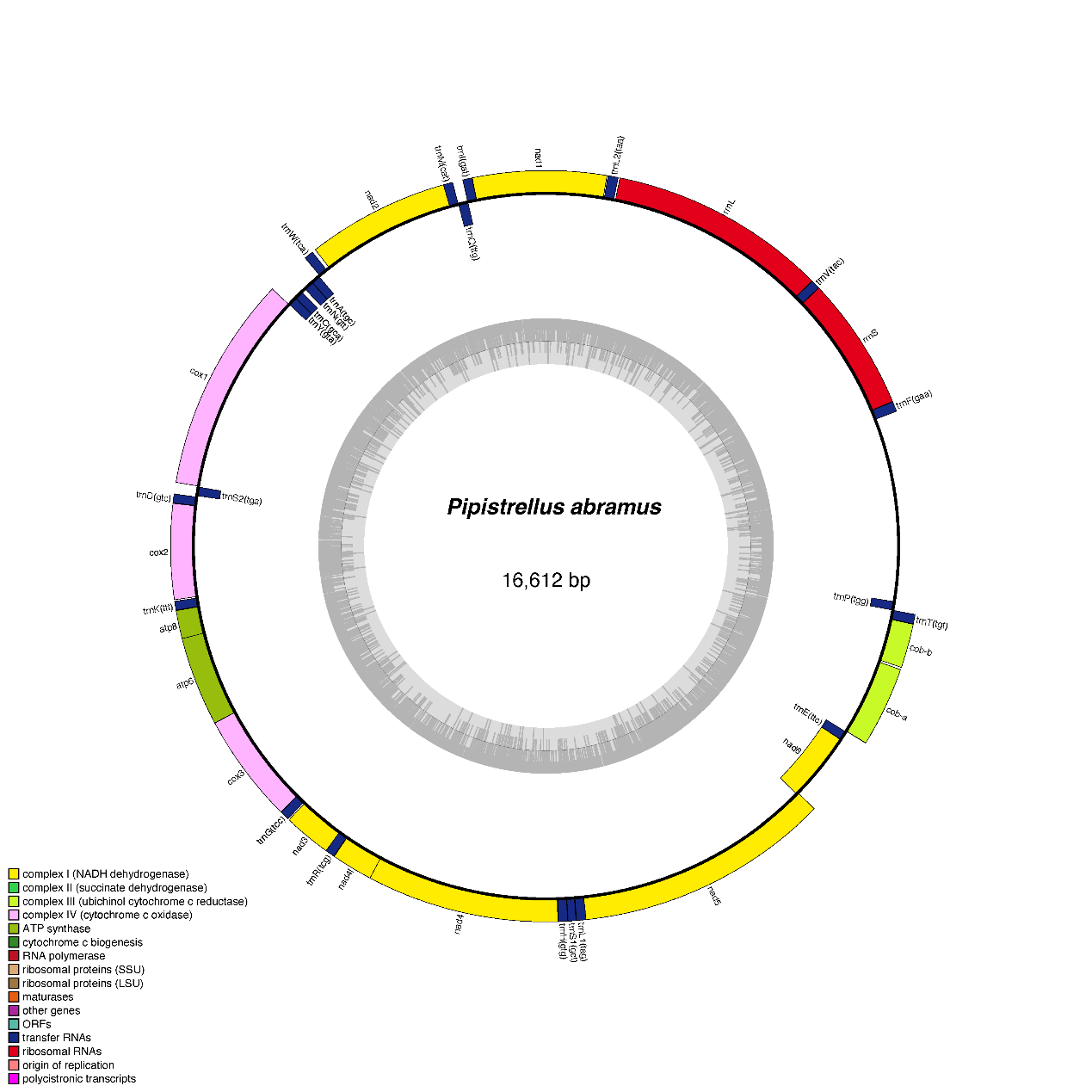


Figure S1. Mitochondrial genome of *P. abramus*.


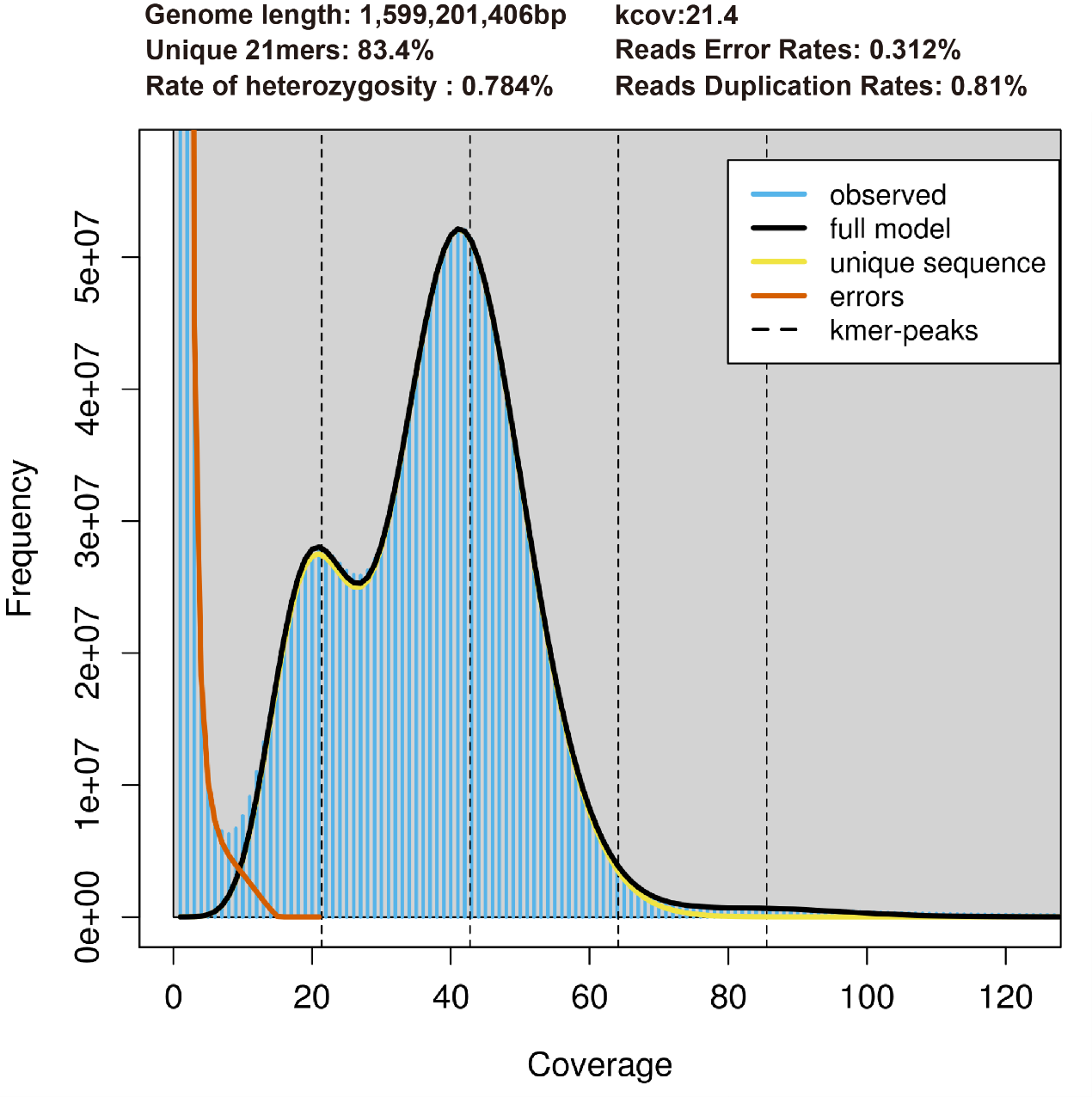


Figure S2. Genomescope profile of *P. abramus* based on Illumina short-read data.


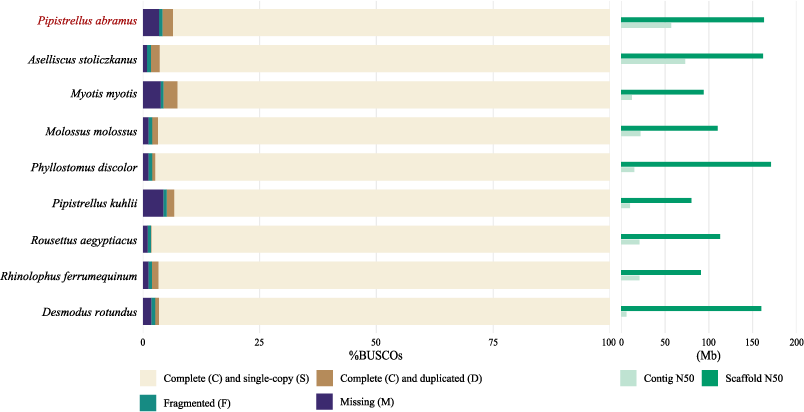


Figure S3. Comparative assessment of genome assembly quality in nine bat species based on BUSCO completeness scores and N50 statistics for Contigs and Scaffolds.


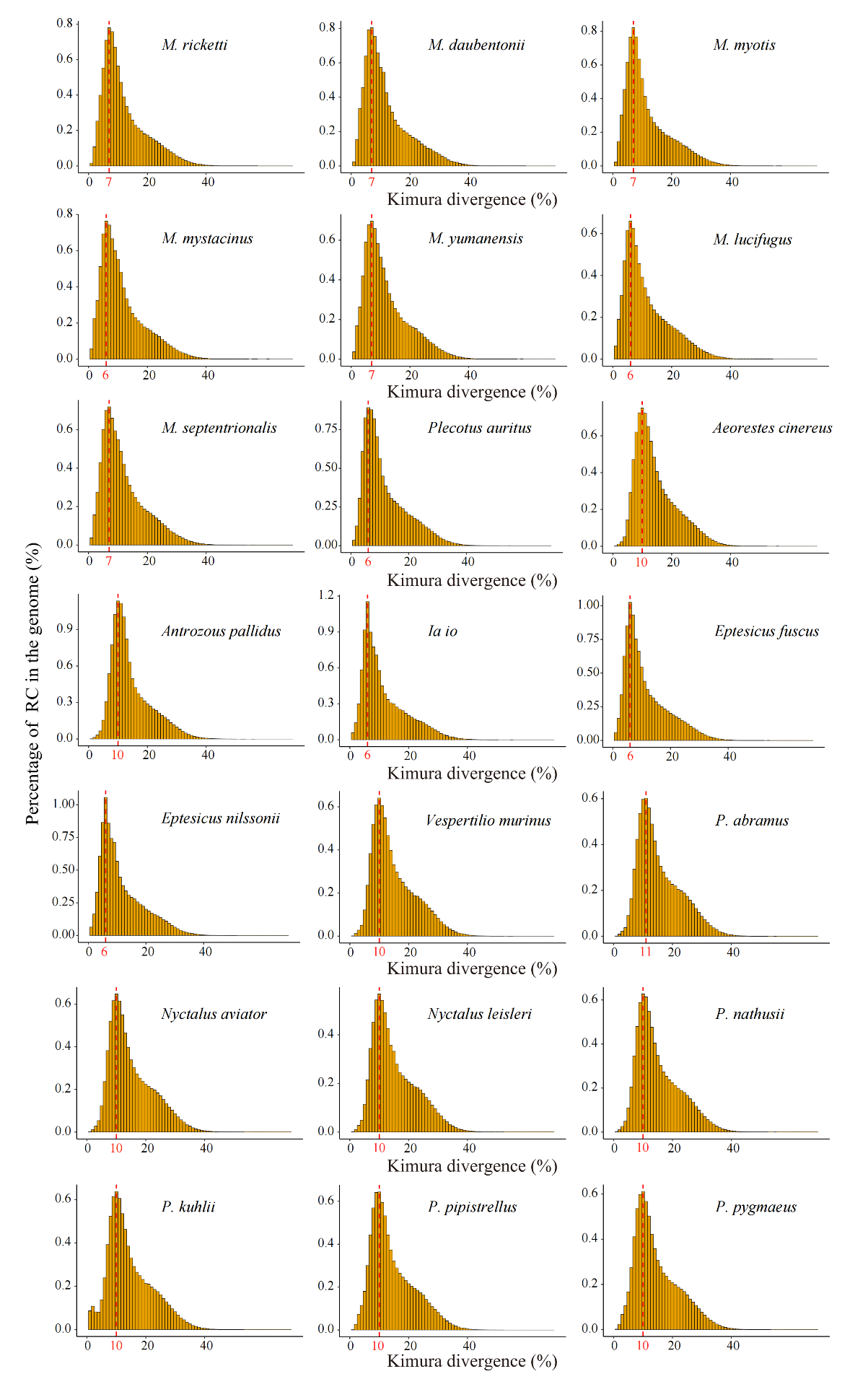


Figure S4. Sequence divergence of rolling-circle (RC) transposons from consensus sequences across genomes of 21 vespertilionid bat species.


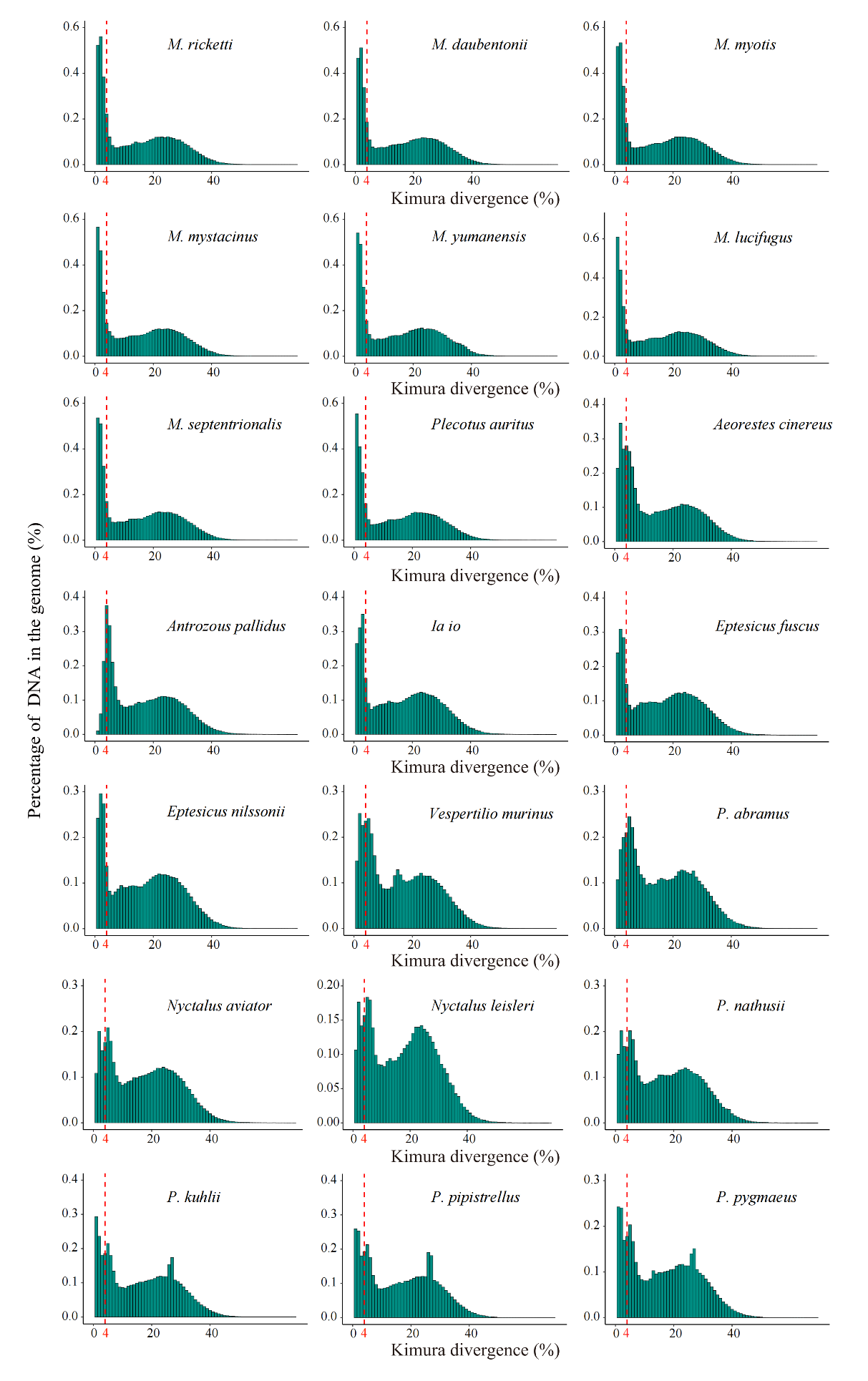


Figure S5. Sequence divergence of DNA transposons from consensus sequences across genomes of 21 vespertilionid bat species.


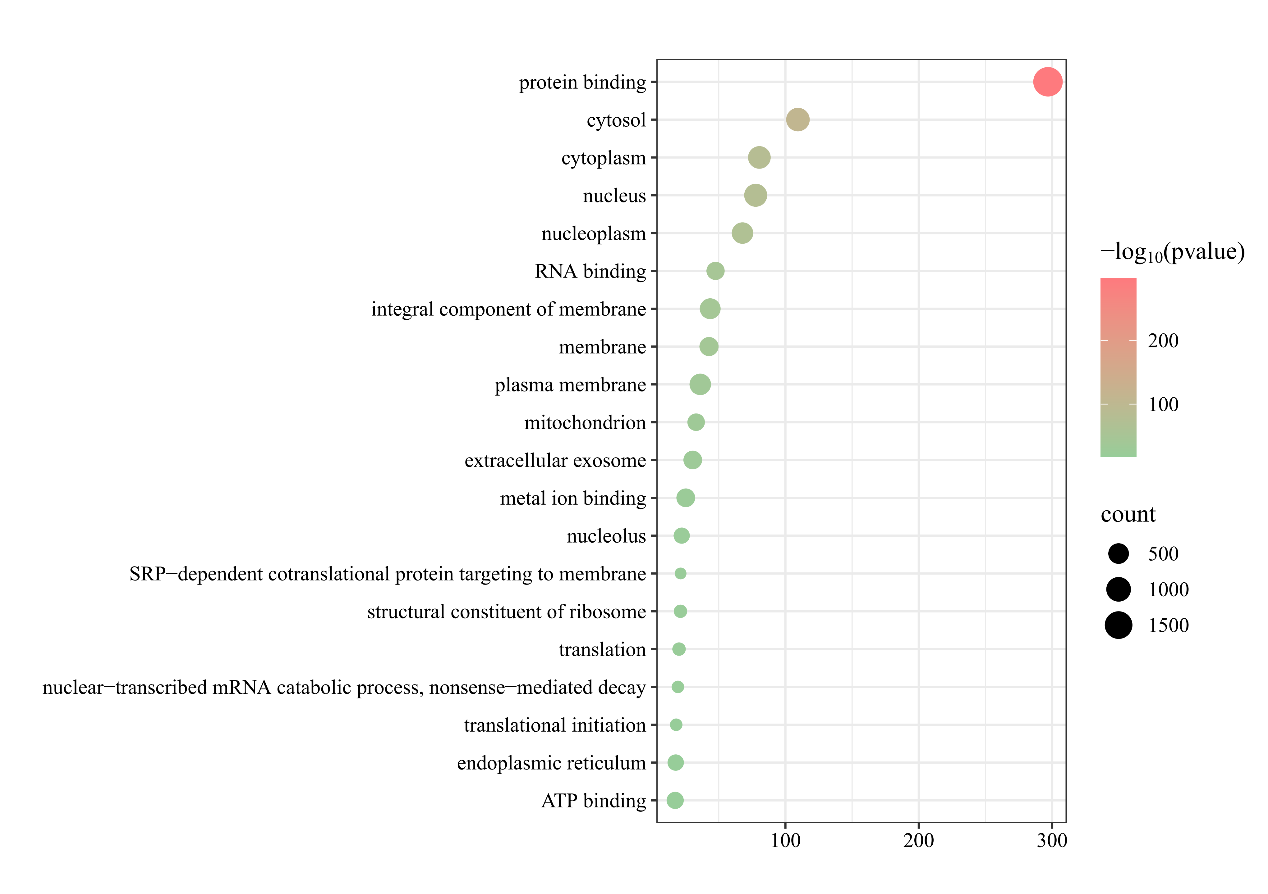


Figure S6. Top 20 significantly enriched GO terms for genes containing RC transposons insertion within their promoter regions in vespertilionid bats.


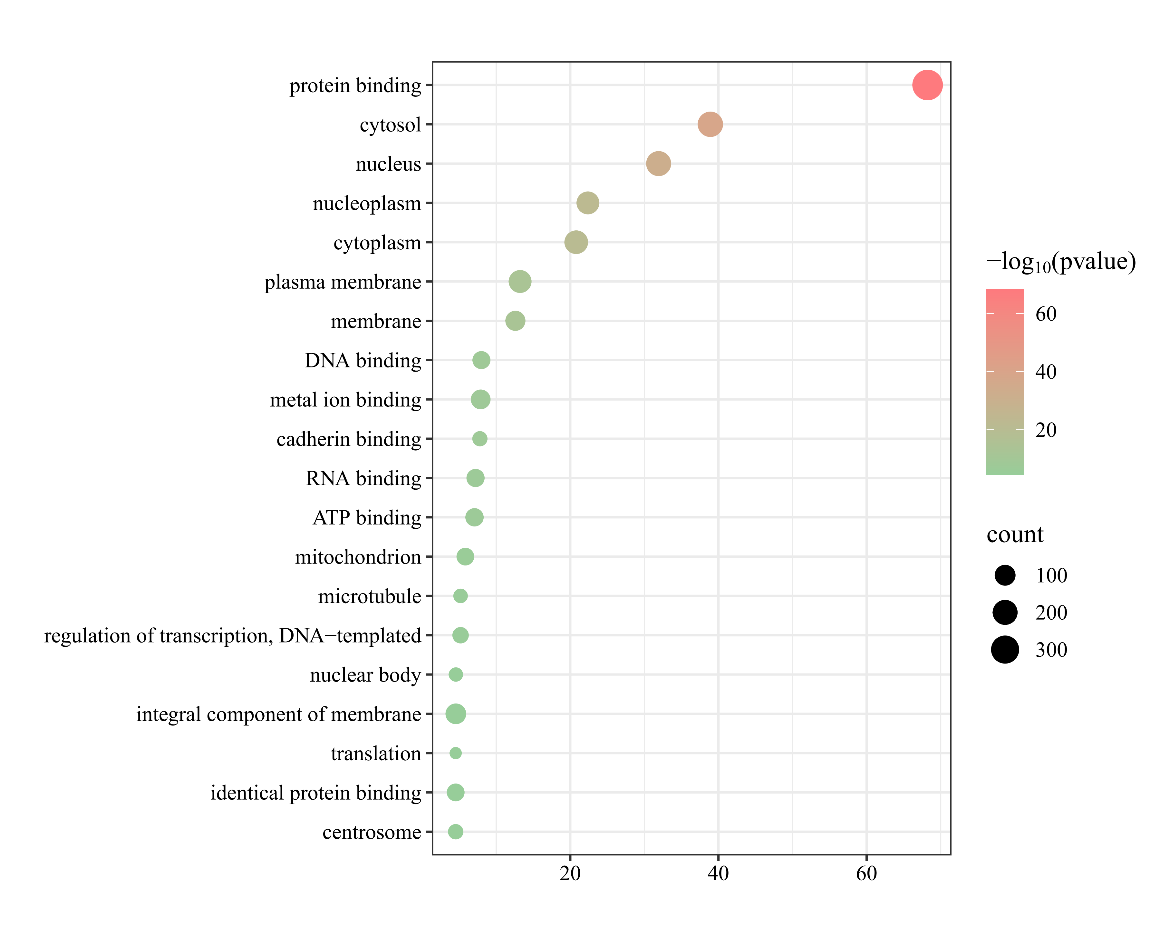


Figure S7. Top 20 significantly enriched GO terms for genes containing RC transposons insertion within their exon regions in vespertilionid bats.


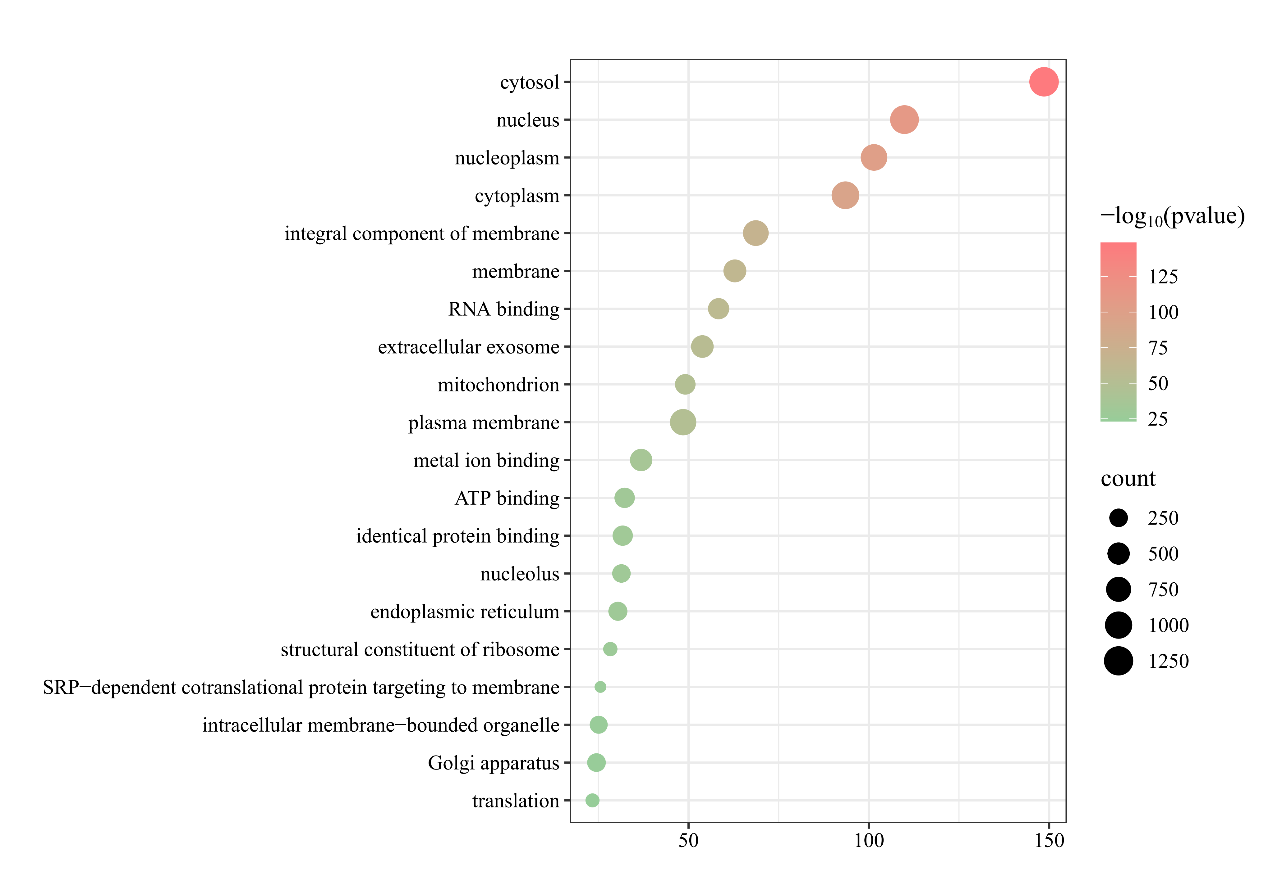


Figure S8. Top 20 significantly enriched GO terms for genes containing insertion of DNA transposons in their promoter regions in vespertilionid bats.


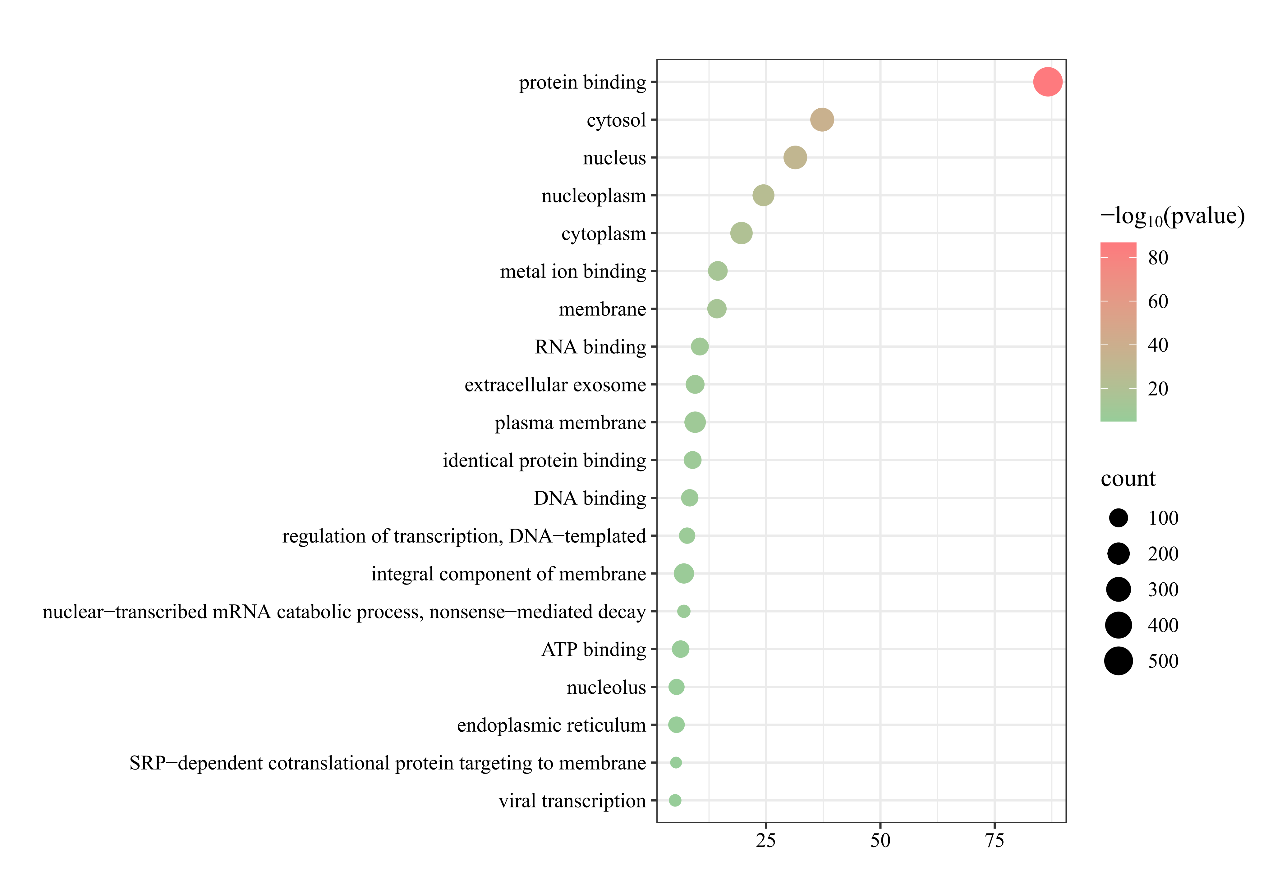


Figure S9. Top 20 significantly enriched GO terms for genes containing insertion of DNA transposons in their the exon regions in vespertilionid bats.


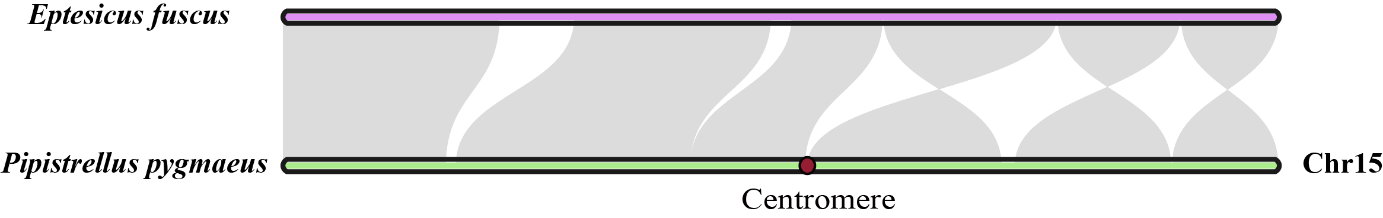


Figure S10. Comparative alignment of the small bi-armed chromosome 15 in *P. pygmaeus* with the homologous acrocentric chromosome in *Eptesicus fuscus*.


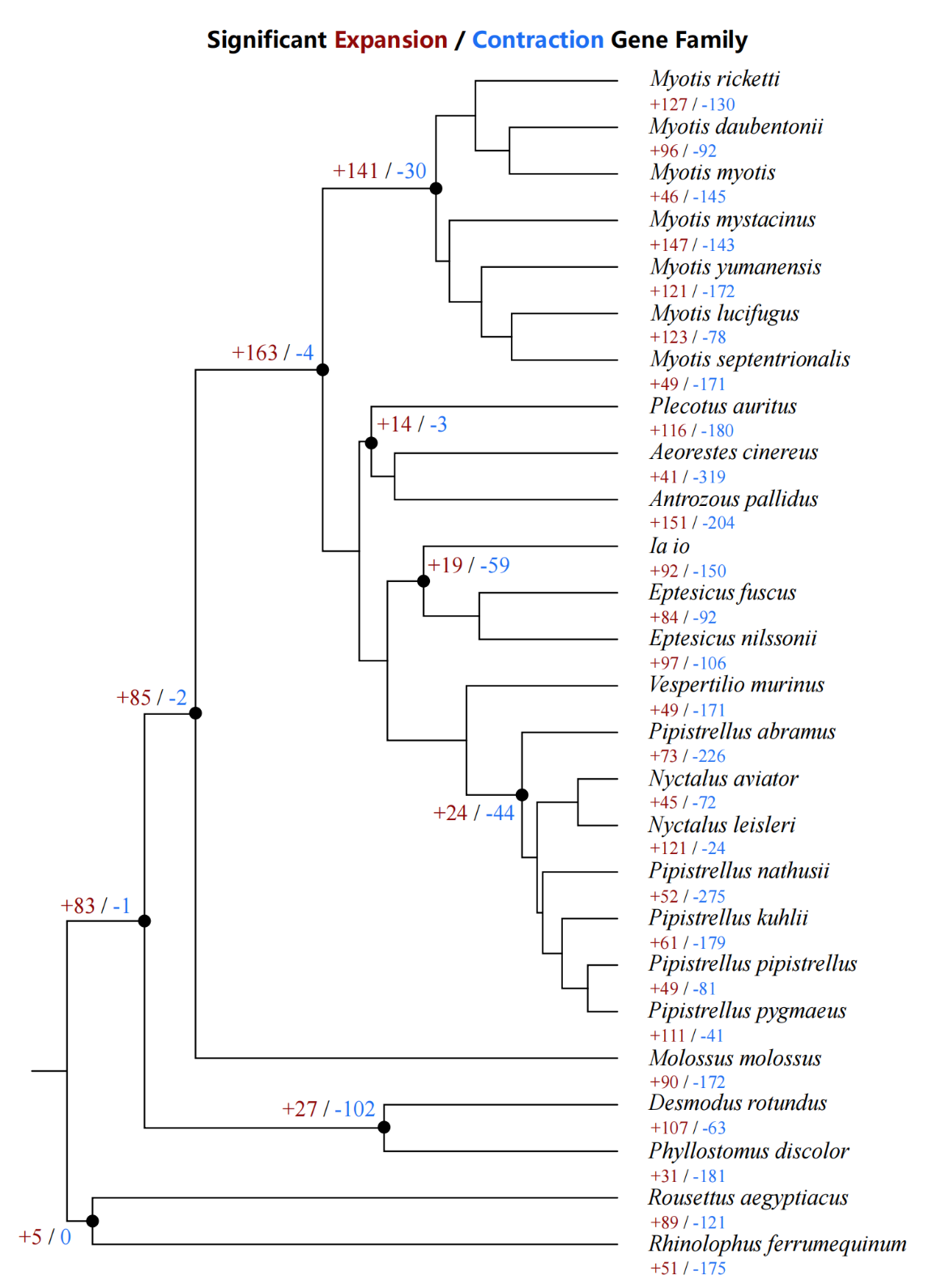


Figure S11. The phylogenetic tree displays counts significantly expanded (red numbers) and contracted (blue numbers) gene families identified for each bat species and evolutionary branch.


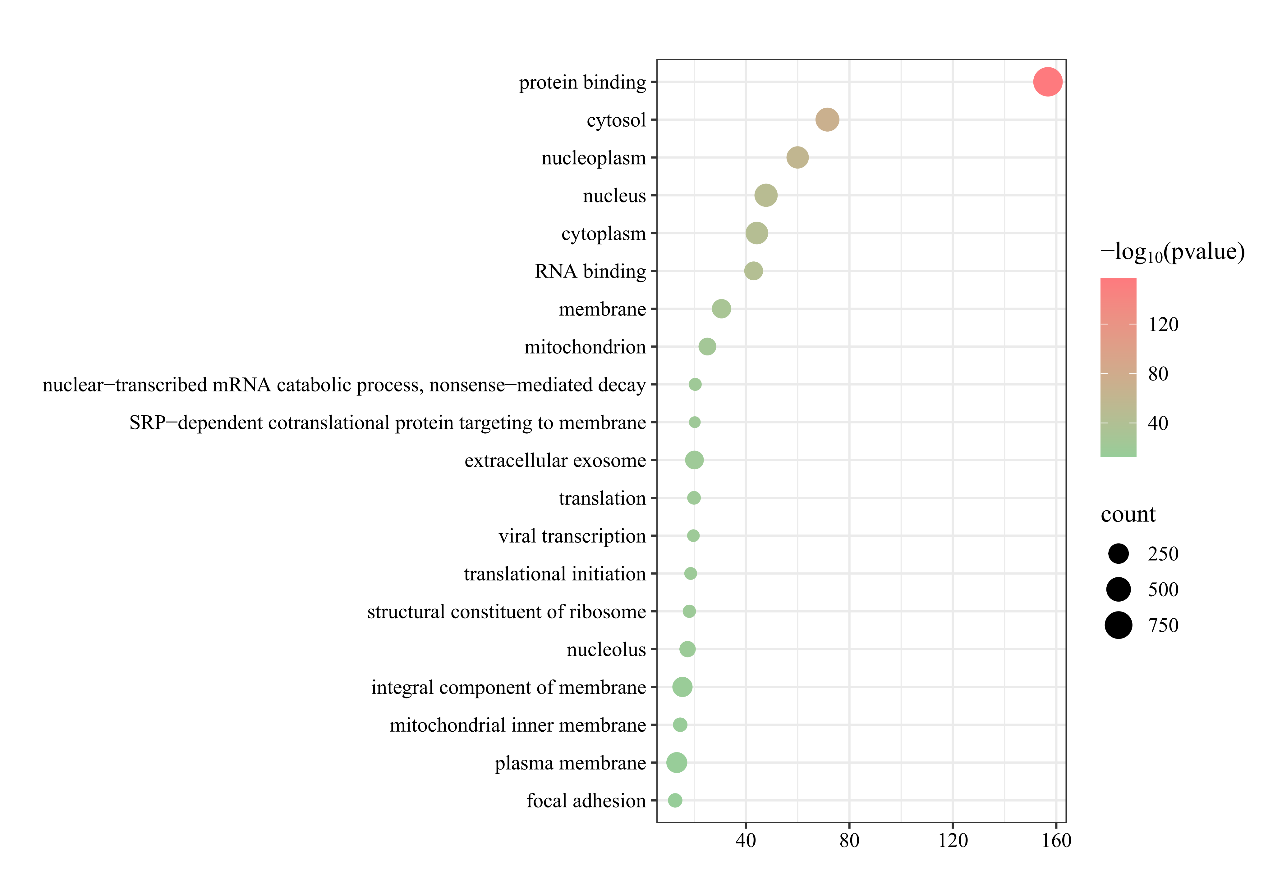


Figure S12. Top 20 significantly enriched GO terms for genes containing insertions of recent SINEs within their promoter regions in *Pipistrellus*.


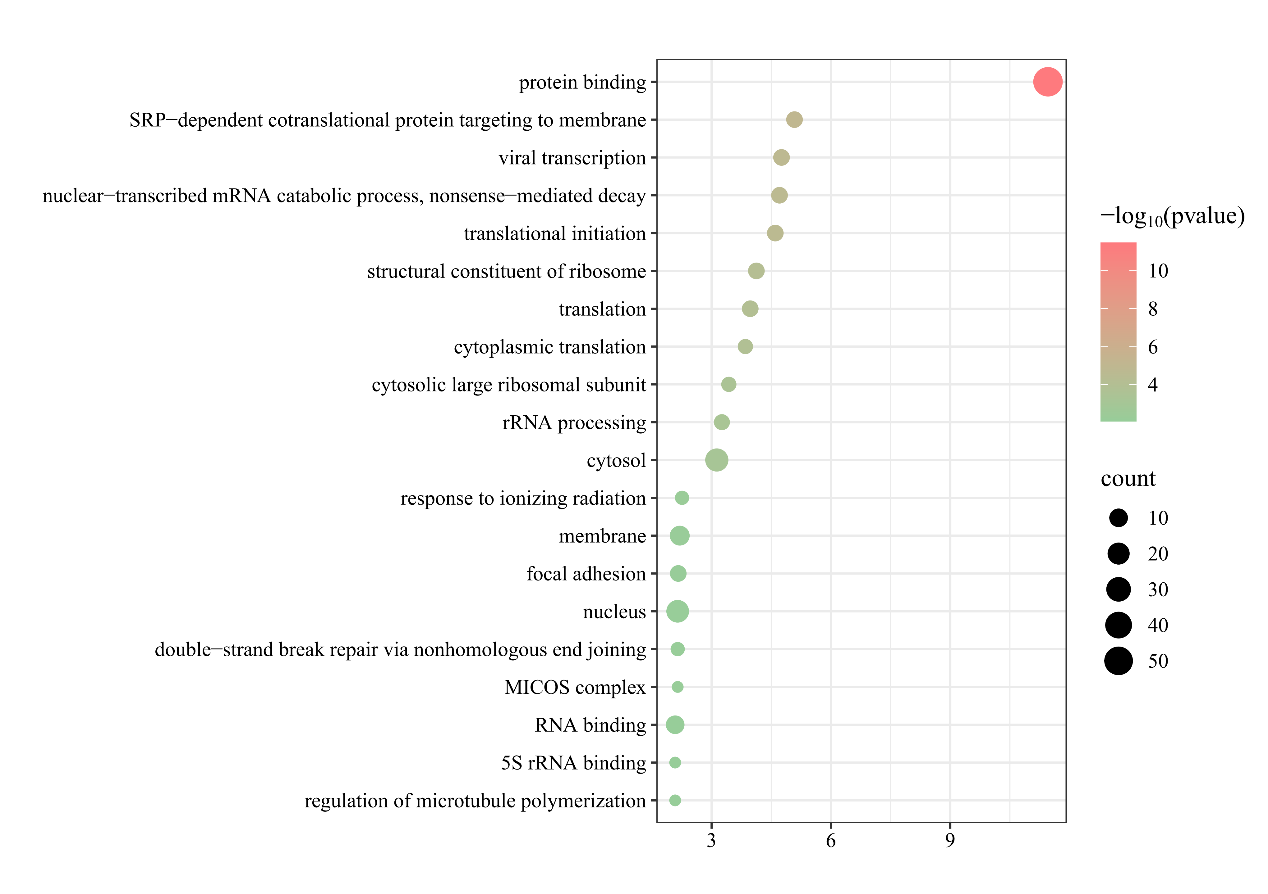


Figure S13. Top 20 significantly enriched GO terms for genes containing insertions of recent SINEs within their exton regions in *Pipistrellus*.
