# Supplementary material for: Comparative Genomics Provide Insights Into Karyotype Evolution in Vespertilionid Bats (Vespertilionidae, Chiroptera)
Source: Mol Ecol Resour. 2026 Mar 20;26(3):e70129. doi: 10.1111/1755-0998.70129 (PMC13003292; doi:10.1111/1755-0998.70129)
Supplement: Supplementary file 2 — Table S1: Summary of genome sequencing and transcriptomic data for P. abramus . Table S2: Information of genome sequences for species used in homology‐based gene prediction. Table S3: Detailed information about the mitogenome annotation for P. abramus . Table S4: Information about genome sequences for 33 species used in phylogenetic reconstruction. Species with available genome annotations were shown in bold. Table S5: Information of chromosome‐level genome assembly for P. abramus . Table S6: Information of the genome assembly and quality assessment for P. abramus . Table S7: Repeat annotation in genome assembly of P. abramus . Table S8: Information of functional annotation and assessment of gene prediction using BUSCOs in P. abramus . Table S9: Reconstructed lengths of ancestral chromosomes (Chr) in the family Vespertilionidae using DESCHRAMBLER. Panels (a) and (b) present chromosome lengths estimates based on reconstructions using one outgroup and five outgroups, respectively. Table S10: Genome sizes of 21 vespertilionid species with chromosome‐level genome assemblies. Table S11: Annotation summary of TEs annotation in 26 bat species. Table S12: Number of genes containing insertions of RC transposons within promoters and exons across 21 vespertilionid species. Table S13: Number of genes containing insertions of DNA transposons within promoters and exons across 21 vespertilionid species. Table S25: Detailed information about the total SINE and recent SINEs (≤ 4% divergence) in vespertilionid bats. Table S28: Length of chromosome fusion sites in chromosomes of P. abramus . Rb: Robertsonian fusion. [file MEN-26-e70129-s003.docx]

Table S1. Summary of genome sequencing and transcriptomic data for *P. abramus*.

a. Statistics of genome sequencing data for *P. abramus*

| Library | Inserted Size（bp） | Number of Clean Reads | Size of clean Reads（Gb） | Average Length（bp） | N50 Length (bp) |
| --- | --- | --- | --- | --- | --- |
|  |  |  |  |  |  |
| Illumina | 350 | 528,493,880 | 79.2 | 150 | / |
| Nanopore | 20,000 | 7,890,425 | 165.55 | 20,981.60 | 32,257 |
| Hi-C | 350 | 1,381,654,922 | 206.44 | 150 | / |

b. Statistics of transcriptome sequencing (RNA-seq) data from eight tissues of *P. abramus*

| Tissue | Number of  Clean Reads | | Size of clean Reads  （bp) |
| --- | --- | --- | --- |
| Brain | 46,174,750 | 6,409,479,254 | |
| Cochlea | 60,248,932 | 8,364,377,380 | |
| Heart | 59,970,868 | 8,345,437,650 | |
| Kidney | 52,965,440 | 7,352,284,334 | |
| Liver | 53,639,198 | 7,458,797,040 | |
| Lung | 57,671,276 | 8,026,436,930 | |
| Spleen | 57,063,324 | 7,944,375,988 | |
| Testis | 52,775,412 | 7,329,850,480 | |

Table S2. Information of genome sequences for species used in homology-based gene prediction.

| **Species** | **RefSeq/GenBank assembly accession** |
| --- | --- |
| *Homo sapiens* | GCF_000001405.40 |
| *Mus musculus* | GCF_000001635.27 |
| *Canis lupus familiaris* | GCF_000002285.5 |
| *Ovis aries* | GCF_016772045.1 |
| *Felis catus* | GCF_018350175.1 |
| *Bos taurus* | GCF_002263795.2 |
| *Myotis lucifugus* | https://www.dnazoo.org/assemblies/myotis_lucifugus |
| *Myotis brandtii* | GCF_000412655.1 |
| *Myotis myotis* | GCF_014108235.1 |
| *Myotis septentrionalis* | https://www.dnazoo.org/assemblies/myotis_septentrionalis |
| *Pipistrellus kuhlii* | GCF_014108245.1 |
| *Rhinolophus ferrumequinum* | GCF_004115265.2 |

Table S3. Detailed information about the mitogenome annotation for *P. abramus*.

| Seq_id | Start | End | Length | Direction | Gene_name |
| --- | --- | --- | --- | --- | --- |
| mitochondrion(circular) | 0 | 74 | 75 | + | trnF(gaa) |
| mitochondrion(circular) | 74 | 1034 | 961 | + | rrnS |
| mitochondrion(circular) | 1034 | 1102 | 69 | + | trnV(tac) |
| mitochondrion(circular) | 1103 | 2661 | 1559 | + | rrnL |
| mitochondrion(circular) | 2671 | 2746 | 76 | + | trnL2(taa) |
| mitochondrion(circular) | 2751 | 3708 | 958 | + | nad1 |
| mitochondrion(circular) | 3707 | 3776 | 70 | + | trnI(gat) |
| mitochondrion(circular) | 3773 | 3847 | 75 | - | trnQ(ttg) |
| mitochondrion(circular) | 3847 | 3916 | 70 | + | trnM(cat) |
| mitochondrion(circular) | 3889 | 4934 | 1046 | + | nad2 |
| mitochondrion(circular) | 4958 | 5026 | 69 | + | trnW(tca) |
| mitochondrion(circular) | 5033 | 5102 | 70 | - | trnA(tgc) |
| mitochondrion(circular) | 5102 | 5175 | 74 | - | trnN(gtt) |
| mitochondrion(circular) | 5177 | 5208 | 32 | + | OL |
| mitochondrion(circular) | 5207 | 5273 | 67 | - | trnC(gca) |
| mitochondrion(circular) | 5273 | 5341 | 69 | - | trnY(gta) |
| mitochondrion(circular) | 5342 | 6887 | 1546 | + | cox1 |
| mitochondrion(circular) | 6891 | 6960 | 70 | - | trnS2(tga) |
| mitochondrion(circular) | 6967 | 7034 | 68 | + | trnD(gtc) |
| mitochondrion(circular) | 7034 | 7718 | 685 | + | cox2 |
| mitochondrion(circular) | 7708 | 7740 | 33 | + | OH_1 |
| mitochondrion(circular) | 7726 | 7792 | 67 | + | trnK(ttt) |
| mitochondrion(circular) | 7793 | 7997 | 205 | + | atp8 |
| mitochondrion(circular) | 7954 | 8635 | 682 | + | atp6 |
| mitochondrion(circular) | 8634 | 9419 | 786 | + | cox3 |
| mitochondrion(circular) | 9418 | 9487 | 70 | + | trnG(tcc) |
| mitochondrion(circular) | 9496 | 9844 | 349 | + | nad3 |
| mitochondrion(circular) | 9834 | 9903 | 70 | + | trnR(tcg) |
| mitochondrion(circular) | 9904 | 10201 | 298 | + | nad4l |
| mitochondrion(circular) | 10194 | 11575 | 1382 | + | nad4 |
| mitochondrion(circular) | 11575 | 11644 | 70 | + | trnH(gtg) |
| mitochondrion(circular) | 11644 | 11703 | 60 | + | trnS1(gct) |
| mitochondrion(circular) | 11704 | 11774 | 71 | + | trnL1(tag) |
| mitochondrion(circular) | 11765 | 13595 | 1831 | + | nad5 |
| mitochondrion(circular) | 13578 | 14106 | 529 | - | nad6 |
| mitochondrion(circular) | 14106 | 14176 | 71 | - | trnE(ttc) |
| mitochondrion(circular) | 14181 | 15321 | 1141 | + | cob |
| mitochondrion(circular) | 15321 | 15391 | 71 | + | trnT(tgt) |
| mitochondrion(circular) | 15390 | 15457 | 68 | - | trnP(tgg) |
| mitochondrion(circular) | 16563 | 16668 | 106 | + | OH_0 |

Table S4. Information about genome sequences for 33 species used in phylogenetic reconstruction. Species with available genome annotations were shown in bold.

| **Species** | **RefSeq/GenBank assembly accession/website** | Complete BUSCOs (%) | Contigs N50 |
| --- | --- | --- | --- |
| *Ia io* | GCA_025583905.1 | 98.1 | 62 Mb |
| ***Eptesicus fuscus*** | GCF_027574615.1 | 98.3 | 49 Mb |
| *Eptesicus nilssonii* | GCA_951640355.1 | 98.2 | 2 Mb |
| *Vespertilio murinus* | GCA_963924515.1 | 97.7 | 3 Mb |
| *Tylonycteris fulvida* | [https://cstr.cn/31253.47.sciencedb.](https://cstr.cn/31253.47.sciencedb.47.85.00)  47.85.00E8FEF7.V3.V4.V5 | 77.9 | 42 Kb |
| *Pipistrellus javanicus* | [https://cstr.cn/31253.47.sciencedb.](https://cstr.cn/31253.47.sciencedb.47.85.00)  47.85.00E8FFF8.V3.V4.V5 | 72.6 | 27 Kb |
| *Nyctalus aviator* | GCA_036971965.2 | 97.8 | 61 Mb |
| *Nyctalus leisleri* | GCA_964264875.1 | 94.8 | 1 Mb |
| *Plecotus auritus* | GCA_963455305.1 | 98.2 | 2 Mb |
| *Pipistrellus nathusii* | GCA_963693515.1 | 97.8 | 17 Mb |
| *Pipistrellus pygmaeus* | GCA_949987585.1 | 97.8 | 54 Mb |
| *Pipistrellus pipistrellus* | GCA_903992545.1 | 92.0 | 4 Mb |
| ***Pipistrellus kuhlii*** | GCF_014108245.1 | 94.9 | 10 Mb |
| *Scotomanes ornatus* | [https://cstr.cn/31253.47.sciencedb.](https://cstr.cn/31253.47.sciencedb.47.85.00)  47.85.00E8FEAA.V3.V4.V5 | 81.3 | 38 Kb |
| *Kerivoula hardwickii* | [https://cstr.cn/31253.47.sciencedb.](https://cstr.cn/31253.47.sciencedb.47.85.00)  47.85.00E9003C.V3.V4.V5 | 84.4 | 66 Kb |
| *Harpiocephalus harpia* | [https://cstr.cn/31253.47.sciencedb.](https://cstr.cn/31253.47.sciencedb.47.85.00)  47.85.00E9014E.V3.V4.V5 | 87.8 | 50 Kb |
| *Murina hilgendorfi* | [https://cstr.cn/31253.47.sciencedb.](https://cstr.cn/31253.47.sciencedb.47.85.00)  47.85.00E8F887.V3.V4.V5 | 94.2 | 78 Kb |
| *Myotis ricketti* | https://doi.org/10.1111/acel.13982 | 98.3 | 79 Mb |
| *Myotis daubentonii* | GCF_963259705.1 | 98.1 | 3 Mb |
| ***Myotis myotis*** | GCF_014108235.1 | 95.6 | 12 Mb |
| *Myotis mystacinus* | GCA_964094495.2 | 95.7 | 2 Mb |
| *Myotis yumanensis* | GCA_028538775.1 | 95.9 | 28 Mb |
| *Scotophilus kuhlii* | [https://cstr.cn/31253.47.sciencedb.](https://cstr.cn/31253.47.sciencedb.47.85.00)  47.85.00E8FDFE.V3.V4.V5 | 77.5 | 39 Kb |
| *Barbastella leucomelas* | [https://cstr.cn/31253.47.sciencedb.](https://cstr.cn/31253.47.sciencedb.47.85.00)  47.85.00E8FE56.V3.V4.V5 | 87.7 | 64 Kb |
| *Aeorestes cinereus* | <https://www.dnazoo.org/>  assemblies/aeorestes_cinereus | 95.4 | 120 Kb |
| *Antrozous pallidus* | GCA_027563665.1 | 97.4 | 41 Mb |
| *Myotis lucifugus* | <https://www.dnazoo.org/>  assemblies/myotis_lucifugus | 91.8 | 64 Kb |
| *Myotis septentrionalis* | <https://www.dnazoo.org/>  assemblies/myotis_septentrionalis | 96.1 | 206 Kb |
| ***Rhinolophus ferrumequinum*** | GCF_004115265.2 | 98.1 | 21 Mb |
| ***Rousettus aegyptiacus*** | GCF_014176215.1 | 98.3 | 21 Mb |
| ***Desmodus rotundus*** | GCF_002940915.1 | 97.4 | 6 Mb |
| ***Phyllostomus discolor*** | GCF_004126475.2 | 98.0 | 15 Mb |
| ***Molossus molossus*** | GCF_014108415.1 | 98.0 | 22 Mb |

Table S5. Information of chromosome-level genome assembly for *P. abramus*.

| Chromosome ID | Length  (bp) | Chromosome ID | Length  (bp) |
| --- | --- | --- | --- |
| Chr1 | 200,938,940 | Chr8 | 126,623,607 |
| Chr2 | 192,850,790 | Chr9 | 103,765,903 |
| Chr3 | 188,058,882 | Chr10 | 84,164,351 |
| Chr4 | 186,846,285 | Chr11 | 52,735,240 |
| Chr5 | 163,267,654 | Chr12 | 37,163,474 |
| Chr6 | 157,497,315 | ChrX | 95,233,838 |
| Chr7 | 154,773,734 | ChrY | 4,358,302 |

Table S6. Information of the genome assembly and quality assessment for *P. abramus*.

|  | Feature | Value |
| --- | --- | --- |
| **Genome assembly statistics** | Total base (bp) of assembly | 1,755,815,725 |
|  | Contigs number | 79 |
|  | Contigs N50 (bp) | 57,073,852 |
|  | Scaffolds number | 48 |
|  | Scaffolds N50 (bp) | 163,267,654 |
|  | GC content (%) | 42.90 |
|  | Hi-C loading rates (%) | 99.57 |
| **Genome assembly evaluation** | Nanopore reads mapping rates (%) | 99.63 |
|  | Illumina reads mapping rates (%) | 99.85 |
|  | RNA-seq reads mapping rates (%) | 95.51 |
| **BUSCO analysis** | Complete BUSCOs (%) | 8,842 (95.8%) |
|  | Complete and single-copy BUSCOs (%) | 8,637 (93.6%) |
|  | Complete and duplicated BUSCOs (%) | 205 (2.2%) |
|  | Fragmented BUSCOs (%) | 63 (0.7%) |
|  | Missing BUSCOs (%) | 321 (3.5%) |
|  | Total BUSCO groups searched | 9,226 |

Table S7. Repeat annotation in genome assembly of *P. abramus*.

| Type of Elements | Number of Elements | Length (bp) | Percentage of Genome (%) |
| --- | --- | --- | --- |
| SINEs | 511,911 | 90,921,757 | 5.18 |
| LINEs | 600,257 | 243,913,334 | 13.89 |
| LTR elements | 317,026 | 82,622,545 | 4.71 |
| DNA transposons | 542,510 | 77,890,017 | 4.44 |
| Rolling-circles | 554,095 | 110,611,838 | 6.30 |
| Unclassified | 92,146 | 14,547,794 | 0.83 |
| **Total interspersed repeats** | 2,617,945 | 620,507,285 | 35.35 |
| Satellites | 5,923 | 715,797 | 0.04 |
| Simple repeats | 665,689 | 34,385,158 | 1.96 |
| Low complexity | 107,073 | 5709,449 | 0.33 |
| **Total tandem repeats** | 778,685 | 40,810,404 | 2.33 |
| **Total** | 3,396,630 | 661,317,689 | 37.68 |

Table S8. Information of functional annotation and assessment of gene prediction using BUSCOs in *P. abramus*.

|  | **Database** | **Number** | **Percentage (%)** |
| --- | --- | --- | --- |
| Functional annotation | Pfam | 18,738 | 79.63% |
|  | SwissProt | 20,539 | 87.28% |
|  | NR | 19,983 | 84.92% |
|  | EggNOG | 20,416 | 86.76% |
|  | All functional annotation | 21,376 | 90.84% |
|  | Unannotated | 2156 | 9.16% |
|  | All | 23,532 | 100% |
|  | **Type** | **Number** | **Percentage (%)** |
| BUSCO analysis | Complete BUSCOs (C) | 9,122 | 98.9 |
|  | Complete and single-copy BUSCOs (S) | 9,038 | 98.0 |
|  | Complete and duplicated BUSCOs (D) | 84 | 0.9 |
|  | Fragmented orthologues | 14 | 0.2 |
|  | Missing orthologues | 90 | 0.9 |
|  | Total BUSCO groups searched | 9,226 | 100 |

Table S9. Reconstructed lengths of ancestral chromosomes (Chr) in the family Vespertilionidae using DESCHRAMBLER. Panels (a) and (b) present chromosome lengths estimates based on reconstructions using one outgroup and five outgroups, respectively.

| Ancestral Chr ID (Chr) | Length (bp)^a^ | Length (bp) ^b^ |
| --- | --- | --- |
| 1 | 194,997,975 | 194,997,975 |
| 2 | 192,770,417 | 192,770,417 |
| 3 | 188,886,325 | 188,886,325 |
| 4 | 102,017,031 | 102,017,031 |
| 5 | 97,150,518 | 97,150,518 |
| 6 | 87,175,415 | 87,175,415 |
| X | 82,850,341 | 82,850,341 |
| 7 | 82,154,476 | 82,154,476 |
| 8 | 74,626,369 | 74,626,369 |
| 9 | 73,165,572 | 73,165,572 |
| 10 | 67,964,974 | 67,964,974 |
| 11 | 65,996,215 | 65,996,215 |
| 12 | 55,002,939 | 55,002,939 |
| 13 | 53,867,905 | 53,867,905 |
| 14 | 53,061,707 | 53,061,707 |
| 15 | 48,597,631 | 48,597,631 |
| 16 | 47,214,416 | 47,214,416 |
| 17 | 42,734,229 | 42,734,229 |
| 18 | 39,396,559 | 39,396,559 |
| 19 | 14,402,604 | 14,402,604 |
| 20 | 13,223,021 | 13,219,037 |
| 21 | 7,802,325 | 8,260,080 |
| total | 1,685,058,964 | 1,685,512,735 |

Table S10. Genome sizes of 21 vespertilionid species with chromosome-level genome assemblies.

| **Species** | **Genome Size (bp)** |
| --- | --- |
| *Ia io* | 2,099,745,439 |
| *Eptesicus fuscus* | 2,008,057,866 |
| *Eptesicus nilssonii* | 2,064,119,045 |
| *Vespertilio murinus* | 1,925,577,803 |
| *Nyctalus aviator* | 1,776,622,221 |
| *Nyctalus leisleri* | 2,233,532,335 |
| *Pipistrellus nathusii* | 1,804,478,338 |
| *Pipistrellus abramus* | 1,755,815,725 |
| *Pipistrellus pipistrellus* | 1,763,439,233 |
| *Pipistrellus pygmaeus* | 1,895,125,685 |
| *Pipistrellus kuhlii* | 1,775,688,541 |
| *Plecotus auritus* | 2,163,221,563 |
| *Myotis ricketti* | 1,998,827,856 |
| *Myotis yumanensis* | 1,952,500,671 |
| *Myotis mystacinus* | 2,081,199,042 |
| *Aeorestes cinereus* | 2,143,505,952 |
| *Antrozous pallidus* | 2,126,460,045 |
| *Myotis daubentonii* | 2,127,824,474 |
| *Myotis myotis* | 2,002,797,769 |
| *Myotis lucifugus* | 2,030,602,833 |
| *Myotis septentrionalis* | 1,977,996,141 |

Table S11. Annotation summary of TEs annotation in 26 bat species.

| Species | SINEs (bp) | LINEs (bp) | LTR elements (bp) | DNA (bp) | Rolling-circles (bp) | Unclassified (bp) | Total (bp) | Percentage of genome (%) |
| --- | --- | --- | --- | --- | --- | --- | --- | --- |
| *Ia io* | 79,405,476  (3.78%) | 356,701,705 (16.99%) | 110,761,314 (5.27%) | 89,827,891 (4.28%) | 185,163,977 (8.82%) | 9,435,238 (0.45%) | 831,295,601 | 39.59 |
| *Eptesicus fuscus* | 79,260,358  (3.95%) | 323,377,489 (16.1%) | 107,457,629 (5.35%) | 84,996,923 (4.23%) | 173,911,191 (8.66%) | 8,969,542 (0.45%) | 777,973,132 | 38.74 |
| *Eptesicus nilssonii* | 79,345,260  (3.84%) | 318,963,696 (15.45%) | 109,319,553 (5.30%) | 85,248,600 (4.13%) | 178,964,151 (8.67%) | 9,748,469 (0.47%) | 781,589,729 | 37.86 |
| *Vespertilio murinus* | 97,093,037  (5.04%) | 272,869,491  (14.17%) | 93,678,569  (4.86%) | 84,564,772  (4.39%) | 124,509,142  (6.47%) | 10,122,460  (0.53%) | 682,837,471 | 35.46 |
| *Nyctalus aviator* | 88,776,612  (5.00%) | 249,527,812  (14.05%) | 86,187,892  (4.85%) | 72,086,266  (4.06%) | 117,488,801  (6.61%) | 7,591,314  (0.43%) | 621,658,697 | 35.00 |
| *Nyctalus leisleri* | 101,393,045  (4.54%) | 302,769,670  (13.56%) | 112,357,818  (5.03%) | 88,799,362  (3.98%) | 133,345,896  (5.97%) | 22,281,980  (1.00%) | 760,947,771 | 34.08 |
| *Pipistrellus nathusii* | 88,288,475  (4.89%) | 255,326,298  (14.15%) | 86,588,827  (4.80%) | 74,612,326  (4.13%) | 117,095,849  (6.49%) | 8,597,950  (0.48%) | 630,509,725 | 34.94 |
| *Pipistrellus abramus* | 90,921,757  (5.18%) | 243,913,334 (13.89%) | 82,622,545  (4.71%) | 77,890,017  (4.44%) | 110,611,838  (6.30%) | 14,547,794  (0.83%) | 620,507,285 | 35.35 |
| *Pipistrellus pipistrellus* | 85,191,488 (4.83%) | 246,236,171 (13.96%) | 87,323,385 (4.95%) | 82,590,115 (4.68%) | 119,702,769 (6.79%) | 7,448,213 (0.42%) | 628,492,141 | 35.63 |
| *Pipistrellus pygmaeus* | 87,818,197 (4.63%) | 248,980,607 (13.14%) | 88,958,303 (4.69%) | 82,493,125 (4.35%) | 121,921,173 (6.43%) | 15,921,129 (0.84%) | 646,092,534 | 34.08 |
| *Pipistrellus kuhlii* | 85,274,187 (4.8%) | 239,355,658 (13.48%) | 85,942,512 (4.84%) | 84,288,834 (4.75%) | 120,842,499 (6.81%) | 6,602,811 (0.37%) | 622,306,501 | 35.05 |
| *Plecotus auritus* | 84,872,731 (3.92%) | 405,799,174 (18.76%) | 119,793,977 (5.54%) | 101,107,665 (4.67%) | 181,539,458 (8.39%) | 10,994,635 (0.51%) | 904,107,640 | 41.79 |
| *Myotis ricketti* | 79,279,288  (3.97%) | 310,013,230  (15.51%) | 125,498,604  (6.28%) | 100,654,813  (5.04%) | 146,927,079  (7.35%) | 8,927,498  (0.45%) | 771,300,512 | 38.60 |
| *Myotis yumanensis* | 78,786,331  (4.04%) | 291,851,338  (14.95%) | 113,102,948  (5.79%) | 95,092,118  (4.87%) | 137,651,248  (7.05%) | 8,622,016  (0.44%) | 725,105,999 | 37.14 |
| *Myotis mystacinus* | 82,292,245  (3.95%) | 335,353,273  (16.11%) | 119,852,337  (5.76%) | 100,177,252  (4.81%) | 154,411,821  (7.42%) | 8,697,891  (0.42%) | 800,784,819 | 38.47 |
| *Aeorestes cinereus* | 78,997,663  (3.69%) | 446,022,110  (20.81%) | 106,368,812  (4.96%) | 93,785,200  (4.38%) | 164,598,251  (7.68%) | 7,096,405  (0.33%) | 896,868,441 | 41.85 |
| *Antrozous pallidus* | 74,033,220  (3.48%) | 386,029,958  (18.15%) | 107,554,891  (5.06%) | 84,768,004  (3.99%) | 218,772,498  (10.29%) | 8,152,169  (0.38%) | 879,310,740 | 41.35 |
| *Myotis daubentonii* | 81,556,441 (3.83%) | 333,876,428 (15.69%) | 127,343,148 (5.98%) | 99,599,100 (4.68%) | 166,314,272 (7.82%) | 10,058,050 (0.47%) | 818,747,439 | 38.47 |
| *Myotis myotis* | 78,586,906 (3.92%) | 319,678,386 (15.96%) | 117,578,239 (5.87%) | 97,479,876 (4.87%) | 145,873,256 (7.28%) | 6,873,510 (0.34%) | 766,070,173 | 38.24 |
| *Myotis lucifugus* | 81,367,344 (4.01%) | 301,266,163 (14.84%) | 116,033,286 (5.71%) | 100,356,200 (4.94%) | 130,523,169 (6.43%) | 8,711,794 (0.43%) | 738,257,956 | 36.36 |
| *Myotis septentrionalis* | 81,491,922 (4.12%) | 296,293,541 (14.98%) | 108,553,918 (5.49%) | 97,586,321 (4.93%) | 143,940,392 (7.28%) | 6,895,780 (0.35%) | 734,761,874 | 37.15 |
| *Molossus molossus* | 158,378,934 (6.84%) | 524,419,004 (22.65%) | 118,468,751 (5.12%) | 73,011,200 (3.15%) | 24,871,901 (1.07%) | 4,322,750 (0.19%) | 903,472,540 | 39.02 |
| *Desmodus rotundus* | 42,724,060 (2.02%) | 444,679,409 (21.04%) | 133,542,396 (6.32%) | 89,990,078 (4.26%) | 9,375,906  (0.44%) | 4,639,572 (0.22%) | 724,951,421 | 34.30 |
| *Phyllostomus discolor* | 57,339,683 (2.74%) | 438,234,443 (20.92%) | 129,445,328 (6.18%) | 88,102,815 (4.21%) | 10,544,881  (0.5%) | 4,266,886 (0.2%) | 727,934,036 | 34.75 |
| *Rhinolophus ferrumequinum* | 56,461,671 (2.72%) | 417,539,335 (20.15%) | 132,969,772 (6.42%) | 110,364,494 (5.33%) | 1,141,694  (0.06%) | 2,899,389 (0.14%) | 721,376,355 | 34.82 |
| *Rousettus aegyptiacus* | 41,420,409 (2.19%) | 335,913,276 (17.74%) | 112,429,238 (5.94%) | 70,844,298 (3.74%) | 1,093,212  (0.06%) | 2,308,045 (0.12%) | 564,008,478 | 29.79 |

Table S12. Number of genes containing insertions of RC transposons within promoters and exons across 21 vespertilionid species.

| **Species** | **Inserted in exons** | **Inserted in promoters** |
| --- | --- | --- |
| *Eptesicus fuscus* | 7 | 162 |
| *Ia io* | 17 | 107 |
| *Myotis daubentonii* | 32 | 179 |
| *Myotis lucifugus* | 73 | 194 |
| *Myotis myotis* | 67 | 164 |
| *Myotis septentrionalis* | 36 | 160 |
| *Pipistrellus abramus* | 21 | 407 |
| *Pipistrellus kuhlii* | 263 | 531 |
| *Pipistrellus pipistrellus* | 56 | 420 |
| *Pipistrellus pygmaeus* | 40 | 410 |
| *Plecotus auritus* | 20 | 122 |
| *Eptesicus nilssonii* | 19 | 124 |
| *Vespertilio murinus* | 24 | 288 |
| *Nyctalus aviator* | 22 | 283 |
| *Nyctalus leisleri* | 29 | 317 |
| *Pipistrellus nathusii* | 11 | 267 |
| *Myotis ricketti* | 22 | 136 |
| *Myotis yumanensis* | 19 | 161 |
| *Myotis mystacinus* | 18 | 114 |
| *Aeorestes cinereus* | 38 | 221 |
| *Antrozous pallidus* | 38 | 304 |

Table S13. Number of genes containing insertions of DNA transposons within promoters and exons across 21 vespertilionid species.

| **Species** | **Inserted in exons** | **Inserted in promoters** |
| --- | --- | --- |
| *Eptesicus fuscus* | 104 | 438 |
| *Ia io* | 29 | 319 |
| *Myotis daubentonii* | 56 | 840 |
| *Myotis lucifugus* | 81 | 935 |
| *Myotis myotis* | 375 | 988 |
| *Myotis septentrionalis* | 76 | 847 |
| *Pipistrellus abramus* | 16 | 292 |
| *Pipistrellus kuhlii* | 65 | 342 |
| *Pipistrellus pipistrellus* | 26 | 326 |
| *Pipistrellus pygmaeus* | 25 | 354 |
| *Plecotus auritus* | 48 | 476 |
| *Eptesicus nilssonii* | 25 | 333 |
| *Vespertilio murinus* | 12 | 300 |
| *Nyctalus aviator* | 12 | 172 |
| *Nyctalus leisleri* | 16 | 169 |
| *Pipistrellus nathusii* | 7 | 157 |
| *Myotis ricketti* | 42 | 738 |
| *Myotis yumanensis* | 53 | 869 |
| *Myotis mystacinus* | 59 | 901 |
| *Aeorestes cinereus* | 44 | 174 |
| *Antrozous pallidus* | 6 | 43 |

Table S25. Detailed information about the total SINE and recent SINEs (≤ 4% divergence) in vespertilionid bats.

| **Species** | Number of recent SINEs | Length (bp) | Number of total SINEs | Length (bp) |
| --- | --- | --- | --- | --- |
| *Eptesicus fuscus* | 10,334 | 2,113,942 | 456,489 | 79,260,358 |
| *Ia io* | 8,697 | 1,766,786 | 458,404 | 79,405,476 |
| *Myotis daubentonii* | 12,305 | 2,522,834 | 479,858 | 81,556,441 |
| *Myotis lucifugus* | 14,198 | 2,862,667 | 468,383 | 81,367,344 |
| *Myotis myotis* | 12,188 | 2,496,326 | 484,739 | 78,586,906 |
| *Myotis septentrionalis* | 16,350 | 3,364,716 | 482,515 | 81,491,922 |
| *Myotis ricketti* | 9,039 | 1,836,017 | 468,466 | 79,279,288 |
| *Myotis yumanensis* | 12,077 | 2,477,632 | 461,083 | 78,786,331 |
| *Myotis mystacinus* | 13,718 | 2,825,924 | 485,219 | 82,292,245 |
| ***Pipistrellus abramus*** | **30,981** | **6,256,383** | **511,911** | **90,921,757** |
| ***Pipistrellus kuhlii*** | **22,631** | **4,524,620** | **484,725** | **85,274,187** |
| ***Pipistrellus pipistrellus*** | **25,712** | **5,161,997** | **481,091** | **85,191,488** |
| ***Pipistrellus pygmaeus*** | **25,956** | **5,211,681** | **493,626** | **87,818,197** |
| ***Nyctalus aviator*** | **27,740** | **5,562,044** | **500,116** | **88,776,612** |
| ***Nyctalus leisleri*** | **34,697** | **6,977,874** | **557,309** | **101,393,045** |
| ***Pipistrellus nathusii*** | **23,476** | **4,688,379** | **497,569** | **88,288,475** |
| *Plecotus auritus* | 11,634 | 2,426,650 | 476,282 | 84,872,731 |
| *Eptesicus nilssonii* | 9,930 | 2,025,621 | 455,922 | 79,345,260 |
| *Vespertilio murinus* | 17,959 | 3,624,093 | 537,185 | 97,093,037 |
| *Aeorestes cinereus* | 5,780 | 1,147,871 | 457,111 | 78,997,663 |
| *Antrozous pallidus* | 567 | 83,909 | 427,560 | 74,033,220 |

Table S28. Length of chromosome fusion sites in chromosomes of *P. abramus*. Rb: [Robertsonian](https://onlinelibrary.wiley.com/doi/abs/10.1002/gcc.20149) fusion

| **Chromosome** | **Start** | **End** | **Length (bp)** | **Fusion Type** |
| --- | --- | --- | --- | --- |
| Chr4 | 102,428,028 | 102,434,446 | 6,418 | Rb |
| Chr5 | 104,362,751 | 104,443,994 | 81,243 | Rb |
| Chr6 | 71,662,673 | 75,095,397 | 3,432,724 | Rb |
| Chr7 | 54,612,389 | 54,783,701 | 171,312 | Rb |
| Chr8 | 53,681,722 | 54,386,630 | 704,908 | Rb |
| Chr9 | 46,165,731 | 47,340,144 | 1,174,413 | Rb |
| Chr9 | 94,526,959 | 94,911,798 | 384,839 | End-to-end |
| Chr10 | 44,458,685 | 44,492,898 | 34,213 | End-to-end |
| Chr12 | 12,610,680 | 12,636,030 | 25,350 | End-to-end |

Table S14. Significantly enriched GO terms for genes containing insertions of RC transposons within their promoter regions in vespertilionid bats.

Table S15. Significantly enriched GO terms for genes containing insertions of RC transposons within their exon regions in vespertilionid bats.

Table S16. Significantly enriched GO terms for genes containing insertions of DNA transposons within their promoter regions in vespertilionid bats.

Table S17. Significantly enriched GO terms for genes containing insertions of DNA transposons within their exon regions in vespertilionid bats.

Table S18. Detailed information about the positively selected genes (PSGs) identified in *Myotis*, Pipistrellini and *P. abramus*.

Table S19. Significantly enriched GO terms for PSGs identified in *P. abramus*.

Table S20. Significantly enriched GO terms for PSGs identified in Pipistrellini.

Table S21. Significantly enriched GO terms for PSGs identified in *Myotis*.

Table S22. Significantly enriched GO terms for genes from expanded/contracted gene families in *P. abramus*.

Table S23. Significantly enriched GO terms for genes from expanded/contracted gene families in Pipistrellini.

Table S24. Significantly enriched GO terms for genes from expanded/contracted gene families in *Myotis*.

Table S26. Genes containing insertions of recent SINEs (≤ 4% divergence) within promoter regions and exon regions in Pipistrellini.

Table S27. Significantly enriched GO terms for genes containing insertions of recent SINEs within exon/promoter regions in Pipistrellini.

Table S29. Annotated genes located in fusion sites of *P. abramus* chromosomes.

Table S30. Significantly enriched GO terms for genes located in fusion sites of *P. abramus* chromosomes.

Table S31. Annotated genes located in flanking regions of fusion sites of *P. abramus* chromosomes.

Table S32. Significantly enriched GO terms for genes located in flanking regions of fusion sites of *P. abramus* chromosomes.
